# The effect of occupational exposure to noise on ischaemic heart disease, stroke and hypertension: A systematic review and meta-analysis from the WHO/ILO Joint Estimates of the Work-Related Burden of Disease and Injury

**DOI:** 10.1016/j.envint.2021.106387

**Published:** 2021-09

**Authors:** Liliane R. Teixeira, Frank Pega, Angel M. Dzhambov, Alicja Bortkiewicz, Denise T. Correa da Silva, Carlos A.F. de Andrade, Elzbieta Gadzicka, Kishor Hadkhale, Sergio Iavicoli, Martha S. Martínez-Silveira, Małgorzata Pawlaczyk-Łuszczyńska, Bruna M. Rondinone, Jadwiga Siedlecka, Antonio Valenti, Diana Gagliardi

**Affiliations:** a Workers' Health and Human Ecology Research Center, National School of Public Health Sergio Arouca, Oswaldo Cruz Foundation, Rio de Janeiro, RJ, Brazil; b Department of Environment, Climate Change and Health, World Health Organization, Geneva, Switzerland; c Department of Hygiene, Faculty of Public Health, Medical University of Plovdiv, Plovdiv, Bulgaria; d Institute for Highway Engineering and Transport Planning, Graz University of Technology, Graz, Austria; e Department of Work Physiology and Ergonomics, Nofer Institute of Occupational Medicine, Lodz, Poland; f Department of Epidemiology and Quantitative Methods in Health, National School of Public Health Sergio Arouca, Oswaldo Cruz Foundation, Rio de Janeiro, RJ, Brazil; g School of Medicine, Universidade de Vassouras, Vassouras, RJ, Brazil; h Faculty of Social Sciences, University of Tampere, Tampere, Finland; i Inail, Department of Occupational and Environmental Medicine, Epidemiology and Hygiene, Monte Porzio Catone, Rome, Italy; j Gonçalo Moniz Institute, Oswaldo Cruz Foundation, Salvador, BA, Brazil; k Department of Physical Hazards, Nofer Institute of Occupational Medicine, Lodz, Poland

**Keywords:** Global burden of disease, Systematic review, Noise, Ischaemic heart disease, Stroke, Hypertension

## Abstract

**Background:**

The World Health Organization (WHO) and the International Labour Organization (ILO) are developing joint estimates of the work-related burden of disease and injury (WHO/ILO Joint Estimates), with contributions from a large number of individual experts. Evidence from mechanistic data suggests that occupational exposure to noise may cause cardiovascular disease (CVD). In this paper, we present a systematic review and meta-analysis of parameters for estimating the number of deaths and disability-adjusted life years from CVD that are attributable to occupational exposure to noise, for the development of the WHO/ILO Joint Estimates.

**Objectives:**

We aimed to systematically review and meta-analyse estimates of the effect of any (high) occupational exposure to noise (≥85 dBA), compared with no (low) occupational exposure to noise (<85 dBA), on the prevalence, incidence and mortality of ischaemic heart disease (IHD), stroke, and hypertension.

**Data sources:**

A protocol was developed and published, applying the Navigation Guide as an organizing systematic review framework where feasible. We searched electronic academic databases for potentially relevant records from published and unpublished studies up to 1 April 2019, including International Trials Register, Ovid MEDLINE, PubMed, Embase, Lilacs, Scopus, Web of Science, and CISDOC. The MEDLINE and Pubmed searches were updated on 31 January 2020. We also searched grey literature databases, Internet search engines and organizational websites; hand-searched reference lists of previous systematic reviews and included study records; and consulted additional experts.

**Study eligibility and criteria:**

We included working-age (≥15 years) workers in the formal and informal economy in any WHO and/or ILO Member State but excluded children (<15 years) and unpaid domestic workers. We included randomized controlled trials, cohort studies, case-control studies and other non-randomized intervention studies with an estimate of the effect of any occupational exposure to noise on CVD prevalence, incidence or mortality, compared with the theoretical minimum risk exposure level (<85 dBA).

**Study appraisal and synthesis methods:**

At least two review authors independently screened titles and abstracts against the eligibility criteria at a first stage and full texts of potentially eligible records at a second stage, followed by extraction of data from qualifying studies. We prioritized evidence from cohort studies and combined relative risk estimates using random-effect meta-analysis. To assess the robustness of findings, we conducted sensitivity analyses (leave-one-out meta-analysis and used as alternative fixed effects and inverse-variance heterogeneity estimators). At least two review authors assessed the risk of bias, quality of evidence and strength of evidence, using Navigation Guide tools and approaches adapted to this project.

**Results:**

Seventeen studies (11 cohort studies, six case-control studies) met the inclusion criteria, comprising a total of 534,688 participants (39,947 or 7.47% females) in 11 countries in three WHO regions (the Americas, Europe, and the Western Pacific). The exposure was generally assessed with dosimetry, sound level meter and/or official or company records. The outcome was most commonly assessed using health records. We are very uncertain (low quality of evidence) about the effect of occupational exposure to noise (≥85 dBA), compared with no occupational exposure to noise (<85 dBA), on: having IHD (0 studies); acquiring IHD (relative risk (RR) 1.29, 95% confidence interval (95% CI) 1.15 to 1.43, two studies, 11,758 participants, I^2^ 0%); dying from IHD (RR 1.03, 95% CI 0.93–1.14, four studies, 198,926 participants, I^2^ 26%); having stroke (0 studies); acquiring stroke (RR 1.11, 95% CI 0.82–1.65, two studies, 170,000 participants, I^2^ 0%); dying from stroke (RR 1.02, 95% CI 0.93–1.12, three studies, 195,539 participants, I^2^ 0%); having hypertension (0 studies); acquiring hypertension (RR 1.07, 95% CI 0.90–1.28, three studies, four estimates, 147,820 participants, I^2^ 52%); and dying from hypertension (0 studies). Data for subgroup analyses were missing. Sensitivity analyses supported the main analyses.

**Conclusions:**

For acquiring IHD, we judged the existing body of evidence from human data to provide “limited evidence of harmfulness”; a positive relationship is observed between exposure and outcome where chance, bias, and confounding cannot be ruled out with reasonable confidence. For all other included outcomes, the bodies of evidence were judged as “inadequate evidence of harmfulness”. Producing estimates for the burden of CVD attributable to occupational exposure to noise appears to not be evidence-based at this time.

**Protocol identifier:**

10.1016/j.envint.2018.09.040.

**PROSPERO registration number:**

CRD42018092272.

## Highlights

• WHO and ILO are developing joint estimates of work-related burden of disease and injury.
• We systematically reviewed effect of occupational exposure to noise on CVD.
• We found 17 eligible studies with 534,688 participants in 11 countries/3 WHO regions.
• We are very uncertain about the effect of occupational exposure to noise on CVD.

## 1 Background

The World Health Organization (WHO) and the International Labour Organization (ILO) are finalizing joint estimates of the work-related burden of disease and injury (WHO/ILO Joint Estimates) (Ryder, 2017). The organizations are estimating the numbers of deaths and disability-adjusted life years (DALYs) that are attributable to selected occupational risk factors. The WHO/ILO Joint Estimates is based on already existing WHO and ILO methodologies for estimating the burden of disease for selected occupational risk factors (International Labour Organization, 2014, Pruss-Ustun et al., 2017). They expand these existing estimates with estimation of the burden of several prioritized additional pairs of occupational risk factors and health outcomes. For this purpose, population attributable fractions (Murray et al., 2004) – the proportional reduction in burden from the health outcome achieved by a reduction of exposure to the risk factor to zero – are being calculated for each additional risk factor-outcome pair. These fractions are being applied to the total disease burden envelopes for the health outcome from the WHO *Global Health Estimates* (World Health Organization, 2017).

The WHO/ILO Joint Estimates may include estimates of the burden of cardiovascular disease (CVD) attributable to occupational exposure to noise, if feasible, as one additional prioritized risk factor-outcome pair. To optimize parameters used in estimation models, the present systematic review and meta-analysis is required of studies with estimates of the effect of occupational exposure to noise on cardiovascular disease (CVD), here defined as comprising prevalence, incidence and mortality of ischaemic heart disease (IHD), stroke, and hypertension (Teixeira et al., 2019). WHO and ILO, supported by a large number of experts, have in parallel also produced a systematic review of studies estimating the prevalence of occupational exposure to noise (Teixeira et al., 2021), applying novel systematic review methods (Pega et al., 2020a). The organizations have conducted or are conducting several other systematic reviews and meta-analyses on other risk factor-outcome pairs (Descatha et al., 2018, Descatha et al., 2020, Godderis et al., 2018, Hulshof et al., 2019, Hulshof et al., 2021b, Li et al., 2018, Li et al., 2020, Mandrioli et al., 2018, Pachito et al., 2020, Paulo et al., 2019, Pega et al., 2020b, Rugulies et al., 2019, Tenkate et al., 2019). To our knowledge, these are the first systematic reviews and meta-analyses (with a pre-published protocol) conducted specifically for an occupational burden of disease study. The WHO/ILO joint estimation methodology and the WHO/ILO Joint Estimates are separate from these systematic reviews, and they will be described and reported elsewhere.

### 1.1 Rationale

To consider the feasibility of estimating the burden of CVD attributable to occupational exposure to noise and to ensure that potential estimates of burden of disease are reported in adherence with the guidelines for accurate and transparent health estimates reporting (GATHER) (Stevens et al., 2016), WHO and ILO require a systematic review of studies on the prevalence of relevant levels of occupational exposure to noise (Teixeira et al., 2021), as well as a systematic review and meta-analysis with estimates of the relative effect of occupational exposure to noise on CVD, compared with the theoretical minimum risk exposure level (presented in this article). The theoretical minimum risk exposure level is the exposure level that would result in the lowest possible population risk, even if it is not feasible to attain this exposure level in practice (Murray et al., 2004). These prevalence and effect estimates should be tailored to serve as parameters for estimating the burden of CVD attributable to occupational exposure to noise in the WHO/ILO Joint Estimates.

We are aware of five previous systematic reviews and/or meta-analyses of studies on the effect of occupational exposure to noise on CVD morbidity and/or mortality. A 2002 systematic review and meta-analysis of 43 studies published between 1970 and 1999 concluded that a 5 dBA increase in noise level was associated with a moderate increase in hypertension risk (relative risk (RR) 1.14, 95% confidence interval (95% CI) 1.01–1.29, 9 studies, I^2^ unclear), but it did not identify any evidence on the effect of occupational noise on other CVD (van Kempen et al., 2002). More recently, three systematic reviews concluded that occupational noise impacts CVD (Domingo-Pueyo et al., 2016, Dzhambov and Dimitrova, 2016, Hwang and Hong, 2012). The Dzhambov and Dimitrova 2016 systematic review found elevated IHD from occupational noise among women, but not among men (Dzhambov and Dimitrova, 2016). A meta-analysis of 12 prospective cohort studies from high-income countries published between 1999 and 2013 (Skogstad et al., 2016) found that exposure to high occupational noise level, generally measured as ≥85 dBA, was associated with a large, clinically meaningful increase in the incidence of hypertension (hazard ratio (HR) 1.68; 95% CI 1.10–2.57, four studies, I^2^ = 88%) and CVD (HR 1.34, 95% CI 1.15–1.56, three studies, I^2^ = 0%), as well as with an increase in the risk of dying from any CVD (HR 1.12; 95% CI 1.02–1.24, five studies, I^2^ = 5%).

To the best of our knowledge, none of the prior systematic reviews on the effect of occupational exposure to noise had a pre-published protocol. Prior systematic reviews did not always adhere to standard requirements outlined in the PRISMA (preferred reporting items for systematic review and meta-analysis) guidelines (Liberati et al., 2009). They did not use two or more reviewers for study selection, data extraction, risk of bias assessment, and/or quality of evidences assessment; did not always specify their eligibility criteria based on PICO (population, intervention, comparator, and outcome) statement or, as promoted in the *Navigation Guide* (Woodruff and Sutton, 2014) PECO (population, intervention, comparator, and outcome); did not always search grey and unpublished literature; and often did not specify key methods (e.g., no search strategy presented and/or data extraction process not described in sufficient detail). Furthermore, the validity of some of their findings has been challenged (Dzhambov and Dimitrova, 2016). Our systematic review is fully compliant with the latest systematic review methods. It builds on previous systematic reviews by covering new evidence up to 31 January 2020.

We emphasize that we also consider workers in both the formal and the informal economy, which may differ in terms of occupational risk factors and exposure effects. The informal economy is defined as “all economic activities by workers and economic units that are – in law or in practice – not covered or insufficiently covered by formal arrangements”, but excluding “illicit activities, in particular the provision of services or the production, sale, possession or use of goods forbidden by law, including the illicit production and trafficking of drugs, the illicit manufacturing of and trafficking in firearms, trafficking in persons and money laundering, as defined in the relevant international treaties” (p. 4) (International Labour Office, 2015).

### 1.2 Description of the risk factor

The definitions of the risk factor, risk factor levels and theoretical minimum risk exposure level are presented in Table 1. Occupational noise is a well-established occupational risk factor (Themann and Masterson, 2019). For investigation of health effects, measures of occupational noise exposure would ideally include information on workers’ activity spaces and patterns of exposure, duration of the exposure, how systematic the exposure is (Guida et al., 2010), sound pressure level and frequency (Branco and Alves-Pereira, 2004), and other relevant risk factors for the health outcome among the exposed population. However, while cumulative occupational exposure to noise may be the most biologically relevant metric from theoretical stance, based on our knowledge of the field and commonly employed approaches to assessment of occupational noise exposure, we believe that global exposure data on agreed cumulative exposure measures do not currently exist. The Global Burden of Disease Study previously classified occupational noise into three categories – minimum exposure (<85 dBA), moderately high exposure (≥85–90 dBA) and high exposure (>90 dBA) (Murray et al., 2004). Presently however, a dichotomized definition is suggested, “Proportion of the population ever exposed to noise greater than 85 dB at work or through their occupation” versus the theoretical minimum risk exposure level being “Background noise exposure” (p. 1362) (GBD 2017 Risk Factor Collaborators, 2018). Hence, here we favoured a more practical dichotomous exposure metric assuming a theoretical minimum risk exposure level of < 85 dBA. Since the theoretical minimum risk exposure level is usually set empirically based on the causal epidemiological evidence, we planned to change the assumed level should evidence suggest an alternative threshold (Teixeira et al., 2019). If several studies consistently reported exposure levels differing from the two standard levels we defined, then, if feasible, we would convert the reported levels to the standard levels; if not, we would report results for these alternative exposure levels as supplementary information in the systematic review (Teixeira et al., 2019).

**Table 1:** Definitions of the risk factor, risk factor levels and the minimum risk exposure level.

Concept	Definition
Risk factor	Occupational noise is the exposure at the workplace to an unpleasant or unwanted sound
Risk factor levels	1. Any occupational exposure to noise (≥85 dBA) 2. No occupational exposure to noise (<85 dBA)
Theoretical minimum risk exposure level	No occupational exposure to noise (<85 dBA)

*Source:*Teixeira et al. (2019).

### 1.3 Definition of the outcome

The WHO *Global Health Estimates* group outcomes into standard burden of disease categories (World Health Organization, 2017), based on standard codes from the International Statistical Classification of Diseases and Related Health Problems, 10th Revision (ICD-10) (World Health Organization, 2015). The relevant WHO *Global Health Estimates* categories for this systematic review are: “*II.H.2 Hypertensive heart disease*”; “*II.H.3 Ischaemic heart disease*”; “*II.H.4 Stroke*”; “*II.H.5 Cardiomyopathy, myocarditis, endocarditis*”; and “*II.H.6 Other circulatory disease*” (World Health Organization, 2017). Table 2 presents WHO *Global Health Estimates* categories and whether they are considered in this systematic review. We planned to exclude from this review cardiovascular abnormalities, cardiovascular infections and pregnancy complications (i.e., ICD-10 codes I01–09; I30; I32–33; I39–43; I47; I49–50; and I52), because an effect of occupational noise on these health outcomes is not yet sufficiently supported by evidence. Therefore, this review covers only a part of the entire disease burden in all five relevant WHO *Global Health Estimates* categories.

**Table 2:** ICD-10 codes and disease and health problems covered by the WHO Global Health Estimates cause categories “II.H.2 Hypertensive heart disease”; “II.H.3 Ischaemic heart disease” and “II.H.4 Stroke” and their inclusion in the systematic review.

ICD-10 code or codes	WHO Global Health Estimates cause category	Included in this review
I10-I15	Hypertensive heart disease	I10–I11, I13–I15
I20-I25	Ischaemic heart disease	I20–I25
I60-I69	Stroke	I60–I69
I30–I33, I38, I40, I42	Cardiomyopathy, myocarditis, endocarditis	I31, I38, I40, I42
I00, I26-I28, I34-I37, I44-I51, I70-I99	Other circulatory diseases	I26–I28, I49, I70–I79

*Source:* Adapted from Teixeira et al. (2019).

### 1.4 How the risk factor may impact the outcome

Official health estimates of the burden of disease attributable to an occupational risk factor require a sufficient level of scientific consensus that the risk factor causes the disease (World Health Organization, 2017). An assessment by WHO of the existing level of evidence on the association between occupational noise and CVD published in 2004 concluded that scientific consensus on causality was insufficient at that point to permit the production of WHO burden of disease estimates (Concha-Barrientos et al., 2004). However, scientists have recently noted that there is now sufficient evidence to reach scientific consensus that environmental noise, including occupational noise, causes CVD (Babisch, 2014, Eriksson et al., 2018a).

**Fig. 1:**
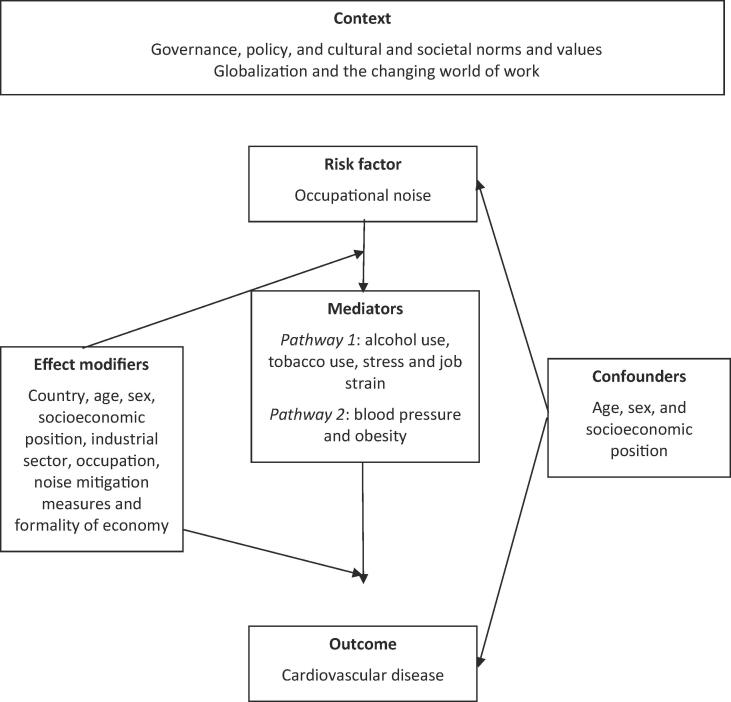
Logic model of the possible causal relationship between occupational exposure to noise and cardiovascular disease.

Fig. 1 presents the logic model for our systematic review of the causal relationship between occupational exposure to noise and CVD. This logic model is an *a priori*, process-oriented one (Rehfuess et al., 2018) that seeks to capture the complexity of the risk factor-outcome causal relationship (Anderson et al., 2011) and is informed by mechanistic evidence on the non-auditory health effects of noise (Babisch, 2014, Münzel et al., 2018, World Health Organization, 2017). Occupational noise may lead to morbidity and mortality from CVD primarily through eliciting an elevated stress response in the organism and promoting vascular damage (Eriksson et al., 2018a). While these mechanisms are not fully understood, there is evidence that several causal pathways operate between occupational noise and CVD. A direct pathway directly links the auditory apparatus to synaptic nervous interactions in the reticular formation and diencephalon, including the hypothalamus, while an indirect pathway involves cognitive processing of sound by cortical and subcortical structures, including the limbic region (Anderson et al., 2011, Andersson and Lindvall, 1988, Recio et al., 2016, Spreng, 2000). Thus, through neuro-endocrine responses (occupational and other) exposure to noise may cause oxidative stress, vascular damage, glucose homeostasis impairment and ultimately CVD (Münzel et al., 2018). These health effects depend on the duration (Guida et al., 2010), repetition (Guida et al., 2010), intensity (Branco and Alves-Pereira, 2004), and frequency of sound exposure (Branco and Alves-Pereira, 2004). In addition, several factors may act as effect modifiers, including individual susceptibility (Job, 1999), ethnicity (Rowland, 1980), sex (Melamed et al., 2004) and other physical (Vangelova and Deyanov, 2007), chemical (Brits et al., 2012, Kirkham et al., 2011, Morata, 1998) and biological risk factors (Brits et al., 2012, Chandola et al., 2010).

As mentioned earlier, noise exposure may have non-auditory effects on living organisms through stress, which leads to vascular damage. This effect has been observed in human studies (Eriksson et al., 2018a). In animal studies usually high (up to 100 dBA) noise intensity levels were applied, which mainly caused direct auditory damage (Münzel et al., 2017). Reviews of the most important research of non-auditory effects of noise in animals were conducted by Turner et al., 2005, Münzel et al., 2017. In the analyzed experiments different exposure conditions were used (noise intensity, characteristics of the sound, duration of exposure, exposure context) and various species of animals were exposed that vary in a hearing ability and physiological response (mice, chinchillas, rabbits, cats, and nonhuman primates). Among non-auditory effects of noise the following have been observed: elevation of blood pressure in cats, rats, rhesus monkeys and macaque monkeys, an increase in the heart rate in desert mule deer and rats, exacerbation in vasoconstriction in rats, an increase in respiratory rates and adrenocorticotropin hormone in cats, elevation of norepinepherine, cortisol, cholesterol, and plasma corticosterone in rats (Turner et al., 2005). Said and El-Gohary (2016) observed many adverse effects on the cardiovascular system (increasing plasma levels of corticosterone, adrenaline, noradrenaline, endothelin-1, nitric oxide and malondialdehyde with a significant decrease in superoxide dismutase plasma levels) in male albino Wistar rats exposed to noise at a level of 80–100 dB. Molina et al. (2016) published a review on noise effects on cell oxidative balance in different tissues, focusing on auditory and non-auditory structures. They concluded that noise exposure can induce extra-auditory effects, mostly in the brain and the immune system, through the generation of an imbalance of the cellular oxidative status.

Münzel et al. (2017) developed a novel noise exposure model in mice (C57Bl/6j), focused on evaluation of the vascular consequences of aircraft noise exposure. In this model, lower exposure parameters (peak sound levels < 85 dBA, mean sound pressure levels 72 dBA) and shorter exposure times (1–4 days) were used. It has been found that such an exposure causes an increase in systolic blood pressure, plasma noradrenaline and angiotensin II concentration, endothelial dysfunction, oxidative stress and inflammation. The newest studies by Steven et al. (2020) in mice (C57BL/6J), exposed for 7 days at a maximum sound pressure level of 85 dB(A) and a mean sound pressure level of 72 dB(A) have shown increased blood pressure, endothelial dysfunction, oxidative stress and inflammation in aortic, cardiac and/or cerebral tissues. The same reaction was observed in mice with experimental arterial hypertension (mice infused with 0.5 mg/kg/d of angiotensin II). In mice subjected to both stressors the effect was enhanced. It should be noted that study models used to date have not reflected occupational noise exposure conditions. Therefore, their results cannot be directly extrapolated to cardiovascular effects in humans occupationally exposed to noise. However, they support the hypothesis about a stress-induced mechanism of noise on CVD development.

## 2 Objectives

To systematically review and meta-analyse evidence on the effect of occupational exposure to noise (≥85 dBA) on CVD prevalence, incidence and mortality among workers of working age, compared with the minimum risk exposure level (<85 dBA).

## 3 Methods

### 3.1 Developed protocol

The *Navigation Guide* (Woodruff and Sutton, 2014) methodology for systematic reviews in environmental and occupational health was used as our guiding methodological framework, wherever feasible. The *Navigation Guide* applies established systematic review methods from clinical medicine, including standard Cochrane Collaboration methods for systematic reviews of interventions, to the field of environmental and occupational health. The methods ensure systematic and rigorous evidence synthesis on environmental and occupational risk factors that reduces bias and maximizes transparency (Woodruff and Sutton, 2014). The need for further methodological development and refinement of the relatively novel Navigation Guide has been acknowledged (Woodruff and Sutton, 2014). Our Systematic Review maps closely to the *Navigation Guide* framework, and steps 1–6 for the stream on human data were conducted, but no steps for the stream on non-human data, although we narratively summarized in brief the evidence from non-human data that we are aware of.

We have registered the protocol in PROSPERO under CRD42018084131, which adheres to the preferred reporting items for systematic review and meta-analysis protocols statement (PRISMA-P) (Moher et al., 2015, Shamseer et al., 2015), with the abstract adhering to the reporting items for systematic reviews in journal and conference abstracts (PRISMA-A) (Beller et al., 2013). Any modification of the methods stated in the protocol was registered in PROSPERO and reported here. The Systematic Review has also been reported according to the PRISMA statement (Liberati et al., 2009). The reporting of the parameters for estimating the burden of CVD from occupational exposure to noise in the systematic review adheres with the requirements of the GATHER guidelines (Stevens et al., 2016), because the WHO/ILO burden of disease estimates that may be produced based on the findings of the systematic review must also adhere to these reporting guidelines.

All methods and reporting guidelines were standardised across all systematic reviews conducted for the WHO/ILO Joint Estimates (Descatha et al., 2018, Descatha et al., 2020, Godderis et al., 2018, Hulshof et al., 2019, Hulshof et al., 2021a, Hulshof et al., 2021b, Li et al., 2018, Li et al., 2020, Mandrioli et al., 2018, Pachito et al., 2020, Paulo et al., 2019, Pega et al., 2020a, Rugulies et al., 2019, Teixeira et al., 2019, Tenkate et al., 2019).

### 3.2 Searched literature

#### 3.2.1 Electronic academic databases

We searched the following electronic academic databases:

1. Ovid MEDLINE (1 January 1946 to 21 March 2019 and updated on 31 January 2020).
2. PubMed (1 January 1946 to 21 March 2019 and updated on 31 January 2020).
3. Embase (1 January 1947 up to 29 March 2019).
4. Web of Science (1 January 1945 up to 29 March 2019).
5. Scopus (1 January 1966 up to 1 April 2019).
6. Lilacs (1 January 1985 up to 1 April 2019).

The Ovid MEDLINE search strategy was presented in the published protocol (Teixeira et al., 2019). We adapted the search syntax to suit grey literature resources. The full search strategies for all databases were revised by an information scientist and are presented in Appendix 1 in the Supplementary data. Searches were performed in electronic databases operated in the English language for most databases and Portuguese and Spanish for Lilacs. When we neared completion of the review, we conducted a top-up search of the MEDLINE and PubMed database on 31 January 2020 to capture the most recent publications (e.g., publications ahead of print). Deviations from the proposed search strategy and the actual search strategy are documented in Section 8.

#### 3.2.2 Electronic grey literature databases

We searched the following electronic resources:

1. CISDOC (up to 1 April 2019).
2. OpenGrey (up to 1 April 2019).
3. GreyLit (up to 1 April 2019).

#### 3.2.3 Internet search engines

We also searched the Google (www.google.com) and Google Scholar (www.google.com/scholar) internet search engines and screened the first 100 hits for potentially relevant records, as was previously done in Cochrane Reviews (Pega et al., 2015, Pega et al., 2017).

#### 3.2.4 Organizational websites

The websites of the seven following international organizations and national government departments were searched:

1. International Labour Organization (www.ilo.org/).
2. World Health Organization (www.who.int).
3. European Agency for Safety and Health at Work (https://osha.europa.eu/en).
4. Eurostat (www.ec.europa.eu/eurostat/web/main/home).
5. China National Knowledge Infrastructure (www.cnki.net/).
6. Finnish Institute of Occupational Health (www.ttl.fi/en/).
7. National Institute of Occupational Safety and Health (NIOSH) of the United States of America, using the NIOSH data and statistics gateway (www.cdc.gov/niosh/data/).

#### 3.2.5 Hand-searching and expert consultation

We hand-searched for potentially eligible studies in:

• Reference lists of previous systematic reviews.
• Reference lists of all included study records.
• Study records published over the past 24 months in the three peer -reviewed academic journals with the largest number of included studies.
• Study records that have cited the included studies (identified in Web of Science citation database).
• Collections of the review authors.

Additional experts were contacted with a list of included studies, with the request to identify potentially eligible additional studies.

### 3.3 Selected studies

Study selection was carried out using the Covidence systematic review software. All study records identified in the search were downloaded and duplicates were identified and deleted. Afterwards, at least two review authors, working in pairs, independently screened titles and abstracts (step 1) and then full texts (step 2) of potentially relevant records. A third review author resolved any disagreements between the first two review authors. If a study record identified in the literature search was authored by an author of this review, the record was assigned to another review author for screening. The study selection is presented in a flow chart, as per PRISMA guidelines (Liberati et al., 2009).

### 3.4 Eligibility criteria

The PECO (Liberati et al., 2009, Morgan et al., 2018) criteria are described below. Our protocol paper provides a complete, but briefer overview of the PECO criteria (see Teixeira et al., 2019 in Appendix A).

#### 3.4.1 Types of populations

We included studies of working-age (≥15 years) workers in formal and informal economy. Studies of children (aged < 15 years) and unpaid domestic workers were excluded. Participants residing in any Member State of WHO and/or ILO and any industrial setting or occupation were included. We note that occupational exposure to noise may potentially have farther population reach (e.g. through the release of noise from the workplace into the community) and acknowledge that the scope of our systematic reviews may not be able capture these populations and impacts on them.

#### 3.4.2 Types of exposures

We included studies that define occupational noise in accordance with our standard definition (Table 1). We included all studies of occupational noise, whether measured objectively (e.g. by means of technology, such as a sound level meter), semi-subjectively, such as studies that used measurements by experts (e.g. scientists with subject matter expertise) or based on self-reports by a worker or workplace administrator or manager. If a study reported both objective and subjective measures, then we prioritized the objective measure. We included studies with measures from any data source, including registry data.

#### 3.4.3 Types of comparators

The comparator considered was participants exposed to the theoretical minimum risk exposure level (Table 1). We excluded all other comparators.

#### 3.4.4 Types of outcomes

This systematic review included nine outcomes:

1. Has IHD (IHD prevalence).
2. Acquired IHD (IHD incidence).
3. Died from IHD (IHD mortality).
4. Has stroke (stroke prevalence).
5. Acquired stroke (stroke incidence).
6. Died from stroke (stroke mortality).
7. Has hypertension (hypertension prevalence).
8. Acquired hypertension (hypertension incidence).
9. Died from hypertension (hypertension mortality).

We included studies that defined CVD in accordance with our standard definition of the eligible outcomes (Table 2). We expected that most studies on occupational exposure to noise and its effect on CVD would have reported ICD-10 diagnostic codes. Otherwise, methods proxying the ICD-10 criteria to ascertain the outcome, such as self-reported physician-diagnosis, were employed (see also section *5.3. Limitations of this systematic review*).

The following measurements of cardiovascular disease were regarded as eligible:

(i) Diagnosis by a physician with imaging.
(ii) Hospital discharge record.
(iii) Other relevant administrative data (e.g. record of sickness absence or disability).
(iv) Registry data of treatment for an eligible cardiovascular disease.
(v) Medically certified cause of death.

All other measures were excluded from this systematic review. Objective (e.g., health records) and subjective (e.g., self-reports) measures of the outcome were eligible. If a study presented both objective and subjective measurements, then we prioritized the objective one.

#### 3.4.5 Types of studies

We included studies that investigated the effect of occupational exposure to noise on cardiovascular disease for any study years and capturing any period of years. Eligible study designs were randomized controlled trials (including parallel-group, cluster, cross-over, and factorial trials), cohort studies (both prospective and retrospective), case-control studies, and other non-randomized intervention studies (including quasi randomized controlled trials, controlled before-after studies, and interrupted time series studies). We considered a broader set of observational study designs than is commonly considered because a recent augmented Cochrane Review of complex interventions identified valuable additional studies using such approach (Arditi et al., 2016). As we were interested in quantifying the risk and not in a qualitative assessment of hazard (Barroga and Kojima, 2013), we excluded all other study designs (e.g. uncontrolled before-and-after, cross-sectional, qualitative, modelling, case and non-original studies).

Records published in any year and any language were considered. However, since the electronic database searches were conducted using English language search terms, only records with a title and/or abstract in English could be retrieved at this initial stage. If a record was written in a language other than those spoken by the authors of this review or those of other reviews in the series (Descatha et al., 2018, Descatha et al., 2020, Godderis et al., 2018, Hulshof et al., 2019, Hulshof et al., 2021b, Hulshof et al., 2021a, Li et al., 2018, Li et al., 2020, Mandrioli et al., 2018, Pachito et al., 2020, Paulo et al., 2019, Pega et al., 2020a, Rugulies et al., 2019, Teixeira et al., 2019, Teixeira et al., 2021, Tenkate et al., 2019), (i.e. Arabic, Bulgarian, Chinese, Danish, Dutch, English, French, Finnish, German, Hungarian, Italian, Japanese, Norwegian, Portuguese, Russian, Spanish, Swedish and Thai), then the record was translated into English. Published and unpublished studies were considered. Of note, studies conducted using unethical practices were excluded (e.g., randomized controlled trials that deliberately exposed humans to a known risk factor to human health).

#### 3.4.6 Types of effect measures

We included measures of the relative effect of high occupational exposure to noise on the risk of having, developing or dying from CVD, compared with the theoretical minimum risk exposure level. Included relative effect measures are relative risk (RR), odds ratio (OR) and hazard ratio (HR) for prevalence, incidence and mortality measures (e.g., developed or died from a cardiovascular disease). To ensure comparability of effect estimates and facilitate meta-analysis, if a cohort study presented an OR, then we planned to convert it into a RR, if possible, using the guidance provided in the Cochrane Handbook for Systematic Reviews of Interventions (Higgins and Green, 2011). Otherwise, we would conduct a sensitivity analysis, excluding the ORs from the respective model, to check their influence on the pooled estimate. If needed, we would calculate effect estimates from raw data but not pool them together with adjusted estimates.

As shown in our logic model (Fig. 1), we a priori considered the following variables to be potential effect modifiers of the effect of occupational exposure to noise on CVD: country, age, sex, socioeconomic position, industrial sector, occupation, noise mitigation measures, and formality of economy. We considered age, sex and socio-economic position to be potential confounders. Potential mediators were tobacco smoking, alcohol use, stress, job strain, blood pressure, and obesity. If a study presented estimates for the effect from two or more alternative models that had been adjusted for different variables, then we systematically prioritized the estimate from the model that we considered best adjusted, applying the lists of confounders and mediators identified in our logic model (Fig. 1). We prioritized estimates from models adjusted for more potential confounders over those from models adjusted for fewer. For example, if a study presented estimates from a crude, unadjusted model (Model A), a model adjusted for one potential confounder (Model B) and a model adjusted for two potential confounders (Model C), then we prioritized the estimate from Model C. If possible, we prioritized effect estimates from more parsimonious models unadjusted for mediators over those from models that adjusted for mediators, because adjustment for mediators can introduce bias (Greenland et al., 2016, Greenland and Pearce, 2015, Wang et al., 2017). For example, if Model A had been adjusted for two confounders and Model B had been adjusted for the same two confounders and a potential mediator, then we chose the estimate from Model A over that from Model B. We planned to prioritize estimates from models that could adjust for time-varying confounders that are at the same time also mediators, such as marginal structural models (Pega et al., 2016), over estimates from models that could only adjust for time varying confounders, such as fixed-effects models (Gunasekara et al., 2014), over estimates from models that could not adjust for time-varying confounding. If a study presented effect estimates from two or more potentially eligible models, then we explained specifically why we prioritized the selected model. In some cases (e.g. Kersten and Backe (2015)) we extracted effect estimates for different subgroups from the same study and treated them as separate data points in the meta-analysis, if they did not share subjects.

### 3.5 Extracted data

A standard data extraction form was developed and trialled until data extractors reached convergence and agreement. At least two review authors independently extracted data on study characteristics (including study authors, study year, study country, participants, exposure and outcome), study design (including study type, comparator, epidemiological model(s) used and effect estimate measure) and risk of bias. A third review author resolved conflicts in data extraction. Data were entered into and managed with Excel.

We also extracted data on potential conflict of interest in included studies. For each author and affiliated organization of each included study record, we extracted their financial disclosures and funding sources. We used a modification of a previous method to identify and assess undisclosed financial interest of authors (Forsyth et al., 2014). Where no financial disclosure or conflict of interest statements were available, we searched the name of all authors in other study records gathered for this study and published in the prior 36 months and in other publicly available declarations of interests (Drazen et al., 2010a, Drazen et al., 2010b).

### 3.6 Requested missing data

Whenever needed, we attempted to contact the corresponding authors of respective publications and requested re-analysis of their data. This was done if the risk estimate was not reported in a suitable format for pooling together with other studies (e.g., a different cut-off exposure level; Pettersson et al. (2020)) or if multiple comparisons were reported within a study (e.g., a single control group and several exposed groups stratified by duration of exposure (e.g. Davies (2002)) (see Appendix 2 in the Supplementary data).

### 3.7 Assessed risk of bias

Standard risk of bias tools do not exist for systematic reviews for risk assessment in occupational and environmental health, nor for risk assessment. The five methods specifically developed for occupational and environmental health are for either or both hazard identification and risk assessment and they differ substantially in the types of studies (randomized, observational and/or simulation studies) and data (e.g. human, animal and/or in vitro) they seek to assess (Rooney et al., 2016). However, all five methods, including the *Navigation Guide* (Lam et al., 2016b), assess risk of bias in human studies similarly (Rooney et al., 2016).

The *Navigation Guide* was specifically developed to translate the rigor and transparency of systematic review methods applied in the clinical sciences to the evidence stream and decision context of environmental health (Woodruff and Sutton, 2014), which includes workplace environment exposures and associated health outcomes. Consistent with using the *Navigation Guide* as our organizing framework, we used its risk of bias tool, which builds on the standard risk of bias assessment methods of the Cochrane Collaboration (Higgins et al., 2011) and the US Agency for Healthcare Research and Quality (Viswanathan et al., 2008). Some further refinements of the *Navigation Guide* method may be warranted (Goodman et al., 2017), but it has been successfully applied in several completed and ongoing systematic reviews (Johnson et al., 2016, Johnson et al., 2014, Koustas et al., 2014, Lam et al., 2014, Lam et al., 2017, Lam et al., 2016a, Lam et al., 2016, Vesterinen et al., 2015). In our application of the *Navigation Guide* method, we drew heavily on one of its latest versions, as presented in the protocol for an ongoing systematic (Lam et al., 2016b).

We assessed risk of bias on the individual study-level and across the body of evidence for each outcome. The nine risk of bias domains included in the *Navigation Guide* method for human studies are: (i) source population representation; (ii) blinding; (iii) exposure assessment; (iv) outcome assessment; (v) confounding; (vi) incomplete outcome data; (vii) selective outcome reporting; (viii) conflict of interest; and (ix) other sources of bias. Risk of bias ratings for all domains were: “low”; “probably low”; “probably high”; “high” or “not applicable” (Lam et al., 2016b). To judge the risk of bias in each domain, we followed instructions developed *a priori*, which were adopted or adapted from an ongoing *Navigation Guide* systematic review (Lam et al., 2016b). The risk of bias at study level was determined by the worst rating in any bias domain for any outcome. For example, a study was assessed as carrying a “probably high” risk of bias in one domain for one outcome and “low” risk of bias in all other domains for the outcome and in all domains for all other outcomes, the study was rated as having a “probably high” risk of bias overall.

All risk of bias assessors jointly trialled the application of the risk of bias criteria until they synchronized their understanding and application of these criteria. At least two study authors independently judged the risk of bias for each study by outcome. Where individual assessments differed, a third author resolved the conflict. In the systematic review, for each included study, we reported our study-level risk of bias assessment by domain in a standard ‘Risk of bias’ table (Higgins et al., 2011). For the entire body of evidence, we presented the study-level risk of bias assessments in a ‘Risk of bias summary’ figure (Higgins et al., 2011).

### 3.8 Synthesised evidence (including conducted meta-analysis)

We conducted separate meta-analyses of the exposure-effect relationship between occupational noise and incidence and mortality of IHD and stroke and hypertension incidence. Studies of different designs were not combined quantitatively. We did not combine unadjusted with adjusted estimates. We only combined studies that we judged to have a minimum acceptable level of adjustment for the core confounders identified (Fig. 1). Given that single case-control studies were included for each outcome (except for IHD incidence for which there were two), our main meta-analyses are based on the included cohort studies. Results of case-control studies are reported as supporting evidence.

If we found two or more studies reporting eligible effect estimates, two or more review authors independently investigated the clinical heterogeneity of the studies in terms of participants (including country, sex, age and industrial sector or occupation), level of risk factor exposure, comparator and outcomes. When effect estimates were homogenous across countries, sexes and age groups, then we combined studies from all of these populations into one pooled effect estimate that could be applied across all combinations of countries, sexes and age groups in the WHO/ILO joint methodology.

If two or more clinically homogenous studies were found to be sufficiently homogenous statistically to be combined in a meta-analysis, we pooled the risk estimates of the studies using the random effects model (DerSimonian and Laird (2015) to account for cross-study heterogeneity (Figueroa, 2014). Statistical heterogeneity was indicated by a significant Cochran’s Q at the p < 0.1 level and quantified using the I^2^ statistic. The I^2^ cut-offs of 25%, 50%, and 75% suggested low, moderate, and high heterogeneity, respectively (Higgins and Thompson, 2002).

Because of the low number of studies (<10) included in each meta-analysis, the power of tests for funnel plot asymmetry (Egger’s method) would be too low to distinguish chance from real asymmetry (Egger et al., 1997). Therefore, to detect publication bias, we employed the Doi plot (Furuya-Kanamori et al., 2018, Furuya-Kanamori et al., 2019). Briefly, it is a variant of the normal quintile versus effect plot using a rank-based measure of precision (Z score), instead of the standard error, which is plotted against the effect size (Furuya-Kanamori et al., 2018). The most precise studies define the midpoint around which results scatter, whereas smaller less precise studies produce an effect size that scatters increasingly widely, and the absolute Z score gradually increases for both smaller and larger effect sizes on either side of that of the precise studies. Doi plot asymmetry was quantified with the Luis Furuya-Kanamori (LFK) index (Furuya-Kanamori et al., 2018, Furuya-Kanamori et al., 2019). The LFK index quantifies the difference between the two areas under the Doi plot, created by the perpendicular line to the X-axis from the effect size with the lowest absolute Z score on the Doi plot (Furuya-Kanamori et al., 2018). A symmetrical, mountain-like Doi plot and LFK index <|1| indicate no asymmetry, LFK index between |1| and |2|, minor asymmetry, and LFK index >|2|, major asymmetry (Furuya-Kanamori et al., 2018). In empirical simulation studies, these methods have demonstrated greater power to detect publication bias with as few as five estimates than P-value driven methods (Furuya-Kanamori et al., 2019).

The final meta-analysis was conducted in RevMan 5.3, but the data for entry into this program was prepared using other recognized statistical analysis programme, such as Stata (version 10.0) and MetaXL v. 5.3 (EpiGear International Pty Ltd, Sunrise Beach, Queensland, Australia).

We should note that some studies (e.g., Davies, 2002, Ising et al., 1997, Kersten and Backe, 2015, Suadicani et al., 2012, Tessier-Sherman et al., 2017 compared two (or more) noise-exposed groups (≥85 dB) with the same unexposed (control) group, producing several non-independent effect estimates, which could not be included in the meta-analysis as if they came from separate studies. In such cases, we computed a composite (average) study-level effect size for the comparison of each exposed group versus the control group, by taking within-study correlation into consideration as suggested in the Cochrane Handbook for Systematic Reviews of Interventions (Higgins and Green, 2011). This method has been employed in a previous Cochrane review (Pasquali et al., 2018) (for detailed calculation notes see Appendix 3 in the Supplementary data). We followed the principles outlined by Borenstein et al. (2009). Noteworthy, computing a composite effect size by the methods described above was not possible for some studies that did not report group sample size (Stokholm et al., 2013a) or reported only one estimate for workers exposed to ≥85 dB (Chang et al., 2013, Eriksson et al., 2018b, Virkkunen et al., 2005).

When quantitative synthesis was not feasible, then we synthesized the study findings narratively and identified the estimates that we judged to be the highest quality evidence available.

### 3.9 Conducted subgroup and sensitivity analysis

Owing to the insufficient data per outcome, we could not conduct stratified or subgroup meta-analysis by WHO region, sex and/or age, or a combination of these, as per the systematic review protocol (Teixeira et al., 2019).

We conducted the following sensitivity analyses:

• We performed leave-one-out meta-analysis to check the robustness of the point estimate upon exclusion of each individual estimate one-at-a-time.
• We also pooled the studies under two alternative estimators, the fixed effects model (Deeks et al., 2001) and the inverse variance heterogeneity (IVhet) model (Doi et al., 2017).

### 3.10 Assessed quality of evidence

We assessed quality of evidence using a modified version of the *Navigation Guide* quality of evidence assessment tool (Lam et al., 2016b). The tool is based on the Grading of Recommendations, Assessment, Development and Evaluations (GRADE) approach (Schünemann et al., 2011) adapted specifically to systematic reviews in occupational and environmental health (Morgan et al., 2016).

At least two review authors assessed quality of evidence for the entire body of evidence by outcome, with any disagreements resolved by a third review author. We adopted the latest Navigation Guide instructions for grading the quality of evidence (Lam et al., 2016b). We graded the quality of the entire body of evidence by outcome, using the three Navigation Guide standard quality of evidence ratings: “high”, “moderate” and “low” (Lam et al., 2016b) (Table 3). Within each of the relevant domains, we rated the concern for the quality of evidence, using the ratings “none”, “serious” and “very serious”. As per *Navigation Guide*, we started at “high” quality of evidence for randomized studies and “moderate” for observational studies. Quality was downgraded for no concern by nil grades (0), for a serious concern by one grade (−1) and for a very serious concern by two grades (−2). We downgraded the quality of evidence for the following five GRADE reasons: (i) risk of bias; (ii) inconsistency; (iii) indirectness; (iv) imprecision (wide 95% CI) and (v) publication bias. We up-graded the quality of evidence for the following other reasons: large effect, dose–response and plausible residual confounding and bias. The definition of “Large effect” (i.e., RR > 1.25 or <0.75) was adopted from the WHO evidence review on environmental noise and CVD (van Kempen et al., 2018). There had to be compelling reasons to upgrade or downgrade. For example, if we had a serious concern for risk of bias in a body of evidence consisting of observational studies (−1), but no other concerns and there were no reasons for upgrading, then we downgraded its quality of evidence by one grade from “moderate” to “low”.

**Table 3:** Interpretation of the GRADE ratings of the overall quality of evidence and the Navigation Guide ratings for strength of evidence evaluation.

GRADE rating for quality of evidence	Interpretation of GRADE rating	Navigation Guide rating for strength of evidence for human evidence	Interpretation of Navigation Guide rating
**High**	There is high confidence that the true effect lies close to that of the estimate of the effect.	**Sufficient evidence of toxicity**	A positive relationship is observed between exposure and outcome where chance, bias, and confounding can be ruled out with reasonable confidence. The available evidence includes results from one or more well-designed, well conducted studies, and the conclusion is unlikely to be strongly affected by the results of future studies.
**Moderate**	There is moderate confidence in the effect estimate: the true effect is likely to be close to the estimate of the effect, but there is a possibility that it is substantially different.	**Limited evidence of toxicity**	A positive relationship is observed between exposure and outcome where chance, bias, and confounding cannot be ruled out with reasonable confidence. Confidence in the relationship is constrained by such factors as: the number, size, or quality of individual studies, or inconsistency of findings across individual studies. As more information becomes available, the observed effect could change, and this change may be large enough to alter the conclusion.
**Low**	The panel’s confidence in the effect estimate is limited: the true effect may be substantially different from the estimate of the effect	**Inadequate evidence of toxicity**	The available evidence is insufficient to assess effects of the exposure. Evidence is insufficient because of: the limited number or size of studies, low quality of individual studies, or inconsistency of findings across individual studies. More information may allow an assessment of effects.
**Very Low**	There is little confidence in the effect estimate: the true effect is likely to be substantially different from the estimate of effect.		

Adapted from Schünemann et al., 2011, Lam et al., 2016.

### 3.11 Assessed strength of evidence

Our systematic review included observational epidemiologic studies of human data only, and no other streams of evidence (e.g. no studies of non-human data). We applied the standard *Navigation Guide* methodology (Lam et al., 2016b) to rate the strength of the evidence, as it allows for rating human and non-human animal studies separately. The rating was based on a combination of four criteria: (i) quality of body of evidence, (ii) direction of effect, (iii) confidence in effect and (iv) other compelling attributes of the data that may influence certainty. The ratings for strength of evidence for the effect of occupational exposure to noise on cardiovascular disease were “sufficient evidence of toxicity/harmfulness”, “limited evidence of toxicity/harmfulness”, “inadequate evidence of toxicity/harmfulness” and “evidence of lack of toxicity/harmfulness” (Table 3).

## 4 Results

### 4.1 Study selection

**Fig. 2:**
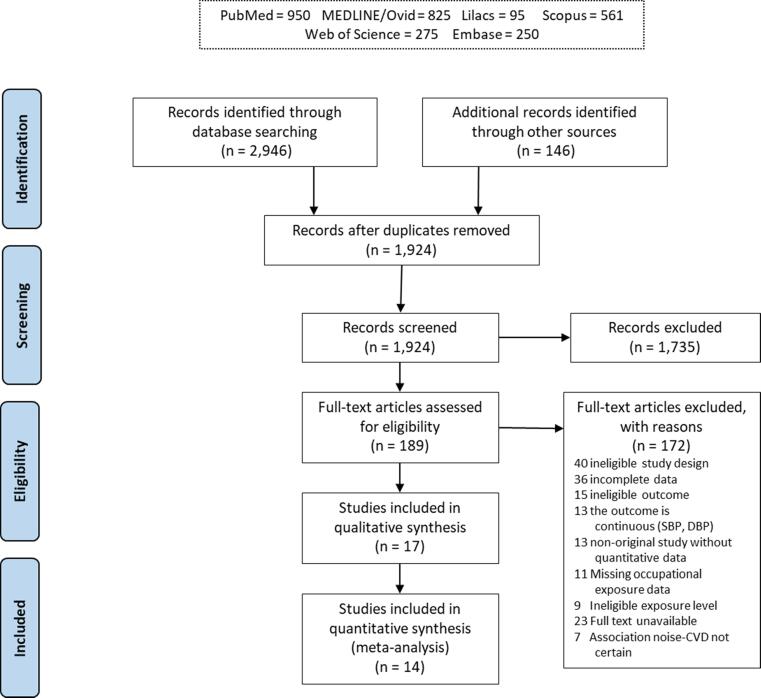
Flow diagram of the study selection. Footnotes: *The study provided deprioritized evidence and was not included in the main meta-analysis due to it being a single case-control study in the respective model (Girard et al., 2015, McNamee et al., 2006, Tong et al., 2017), unadjusted estimate extracted (Huo Yung Kai et al., 2018) or incomparable noise metric (Song, 2013).

A flow diagram of the study selection is presented in Fig. 2. Of the total 3092 individual study records identified in our searches, only 1924 remained after exclusion of duplicities. Of these, 189 records were assessed by full text for eligibility. Only 16 studies fulfilled the eligibility criteria and were included in the systematic review. For the 172 excluded studies that most closely resembled inclusion criteria, the reasons for exclusion are listed in Appendix 4 in the Supplementary data. After updating the search on January 31st 2020, one additional study, which met the inclusion criteria, was added to the list of included studies (Pettersson et al., 2020). Of the 17 included studies in the systematic review, 14 were included in one or more quantitative meta-analyses.

### 4.2 Characteristics of included studies

The characteristics of the included studies are summarized in Table 4.

**Table 4:** Characteristics of included studies (Part I: study population and study type).

Study	Study population							Study type		
Study ID	Total number of study participants	Number of female study participants	Country of study population	Geographic location (specify as 'national' or list regions or sites)	Industrial sector (specify ISIC-4 code provided in worksheet “Industrial sector codes”)	Occupation (specify ISCO-08 code provided in worksheet “Occupation code”)	Age	Study design	Study period (month of first collection of any data and month of last collection of any data)	Follow-up period (period in months between exposure and outcome)
Chang et al. (2013)	578	0	Taiwan	Local	30	7232	27.7 ± 5.3 years	Cohort study (prospective)	1998–2008	9.8 ± 5.2 years
Davies (2002)	27,499 (this is the analysis sample)	0	Canada	Local	16	8172	Mean 29,7 years, range 10,6–76,3 years	Cohort study (retrospective)	1950–1995	Mean 24,3 years, range 1–46 years
Eriksson et al. (2018b)	Baseline-5,753	0	Sweden	Local	Unclear	Unclear	Baseline 55.3 (2.1) years, range 50–59 years	Cohort study (prospective)	1974–1977; 2004 (last noise exposure data)	Unclear
Girard et al. (2015)*	644	0	Canada	Regional	25	Unclear	55–64 years (cases mean = 60.0, controls mean = 58.8)	Case-control	1983–2005/07	Cases - mean: 31.9 years, controls: 29.8 years, all study subjects: 30.5 years
Gopinath et al. (2011)	Blue Mountains Eye Study (BMES-1) 1992–4 – 3,654 participants BEMS-2 (1997–1999) −3,509 participants; BEMS-3 (2002–2004)-1,952 participants	Unclear (BMES-1) 1992–4 – controls: 1,348 females, Exposed: 306 females BMES −2 –no data about females BMES-3 1,556 participants-917 females	Australia	National	No data	No data	67.9 ± 9.4 years (unexposed group) and 67.1 ± 8.9 (exposed)	Cohort study	Baseline: 1997–1999 -incidence study, 2002–2004 - mortality study, cut off point for CHD and stroke death -end of December 2007	Prevalence data was obtained from BMES-2 (baseline), while, incidence analyses used data obtained from both BMES-2 and 5-year follow-up examination (BMES-3).
Huo Yung Kai et al. (2018)*	1,156	About 547	France	National	No data	No data	32 years, 42 years, 52–62 years	Cohort study (prospective)	2001–2006	5 years
Ising et al. (1997)	2,543	0	Germany	Local	No data	No data	31–70 years	Case-control study	Unclear	No data
Kersten and Backe (2015)	4,113	1,059	Germany	Regional	Unclear	1111, 1112, 1321, 1324, 1330, 1420, 2149, 2263, 2269, 2351, 2359, 2433, 4221, 4323, 6113, 7223, 7233, 7322, 7549, 8111, 8121, 9214, 9216	20–69 years	Case-control study	No data	N/A
McNamee et al. (2006)*	1,220	0	United Kingdom	Regional	35	7131	≤75 years	Case-control	1965–1998	≤1 month to ≥40 years
Pettersson et al. (2020)	166,088 (analysis sample)	0	Sweden	National	41–43	No data	15–67 years	Cohort study	1971–1993	17–40 years (ended in 2010)
Song (2013)*	221 cases and 1,105 controls	106 cases and 530 controls	Canada	National	01	1221	< 30 to > 55 years	Case-control	31.12.2001–31.12.2009	180
Stokholm et al. (2013a)	145,190	36,788	Denmark	Regional	1–4; 7–9	8160, 7322, 8112, 8121,8122, 8211, 7231, 8172, 1323, 4419, 7549,8219, 1120	<25 years 25–34 years 35–44 years 45–54 years 55–64 years ≥65 years	Cohort study (prospective)	2001–2007	7 years
Stokholm et al. (2013b)	164,247	Unclear	Denmark	Regional	1–4; 7–9	8160, 7322, 8112, 8121,8122, 8211, 7231, 8172, 1323, 4419, 7549,8219, 1120	Unclear	Cohort study (prospective)	2001–2007	7 years
Suadicani et al. (2012)	5,249 (in 1970) 3,387 (in 1985–1986)	0	Denmark	National	32, 43	3115, 8211, 8121, 8311, 2163, 5112	62.7 (5.2) years	A follow-up study to a cross-sectional survey	1970–1986	16 years
Tessier-Sherman et al. (2017)	2,052	0	USA	Unclear	Section B Mining and quarrying: 24 Manufacture of basic metals, 32 Other manufacturing	8121	Mean 35.8, SD 8.5	Cohort study (retrospective)	After 1 January 1996 to 31 December 2012	72 months; follow-up time - mean 6.5 years
Tong et al. (2017)*	935	0	China	Regional	Iron and steel enterprise (cold rolling and gas factory)	Unclear	≤ 55 years, Essential Hypertension Group − 38.44 ± 8.51 years; Control Group − 38.11 ± 8.04 years	Case-control	February 2014 to July 2014	No follow-up
Virkkunen et al. (2005)	6,005	0	Finland	National	Iron and metal work, machine work in plants, woodworking, and chemical process work	Unclear	40–56 years at entry	Cohort study (prospective)	1982–1999	15.9 years

**Study**	**Exposure assessment**							**Comparator**
Study ID	Exposure definition (i.e. how was the exposure defined?)	Unit for which exposure was assessed	Mode of exposure data collection	Exposure assessment methods	Levels/intensity of exposure (specify unit)	Number of study participants in exposed group	Number of study participants in unexposed group	Definition of comparator (define comparator group, including specific level of exposure)
Chang et al. (2013)	8-hour time-weighted average equivalent sound level with and without adjustment for usage of HPDs (in dBA)	Individual	Technical device	Measurements and questionnaire on HPDs use	< 80 dBA; 80-85 dBA; ≥85 dBA (used in our analyses)	205 (< 80 dBA) 221 (80 to <85 dBA) 152 (≥85 dBA)	205	< 80 dBA (low exposure group)
Davies (2002)	Duration of exposure to noise levels exceeding a specific threshold in L_eq_ dBA (used in meta-analysis); and Cumulative exposure in dBA-year	Individual	Historical exposure levels were estimated by a determinants of exposure regression model, developed using 1,900 personal dosimetry measurements	A combination of measurements, interviews, hygienists' assessment and modelling	For duration of exposure: <3 years (reference), 3–10 years, 10–20 years, 20–30 years, > 30 years for the thresholds > 85 dBA; (≥85 dBA for > 3 years was used for our analyses)	N/A	N/A	Exposure to <85 dBA for < 3 years
Eriksson et al. (2018b)	To assess occupational noise exposure, a previously developed job-exposure matrix was applied	Group level: 129 unique job families	Job-exposure matrix	Measurements reports	<75dBA; 75–85 dBA; >85 dBA (used in our analyses)	2,823	2,930	Exposure to noise: medium < 75 dBA
Girard et al. (2015)*	Exposure to ≥ 90 dBA	Individual	Technical device	Measurements	Exposure to ≥ 90 dBA for < 27, 27–36.4, ≥ 36.5 years; Noise levels ≥90 dBA/8h, cases − 46%, control − 50.9% (used in our analyses)	320	324	< 90 dBA/8h
Gopinath et al. (2011)	Questionnaires on workplace noise exposure history	Individual	Questionnaire	Self-reported	Self-reported exposed status; duration of exposure: 0 years, <1–5 years, >5 years; severity of exposure: none, tolerable, unable to hear speech (used in our analyses)	2,796	1,859	Answer “No” to the question: “Have you ever worked in the noisy industry or noisy farm environment?”
Huo Yung Kai et al. (2018)*	The questions used were similar to those used in the 5th European survey on working conditions in the ESTEV study and in the previous VISAT articles	Individual	Data from French prospective VISAT study	Self-reported	Exposed at baseline or in the preceding five years to (cannot hear a person who is 2–3 m away even if talking loudly)	483	673	Answer “No” to a question on occupational exposure to “loud” noise
Ising et al. (1997)	Subjective noise categories: 1+2 Refrigerator and typewriter 3. electric lawn-mower 4. electric drill 5. pneumatic drill	Work noise level measured as 1-min mean level in relation to the subjective work noise category	Subjective evaluation of noise loudness based on questionnaire	Self-reported and objective measurement in the sample of 80 men using Norsonic Type 110	Subjective noise categories Lower categories (1+2), higher categories (3+4+5) - these noise categories correspond to the median (25 percentiles) of LAeq,T>70 dBA.	395	2,148	Low-noise-exposed workers (noise categories: refrigerator+typewriter)
Kersten and Backe (2015)	Occupational noise (LEX,8h,subj) and (LEX,8h,obj) >55 dBA	Individual	Questionnaire, technical device, and experts judgements	Self-reported vocal effort and equipment catalogue specifications	46–61 dBA, 62–84 dBA, 85–94 dBA, 95–124 dBA	1,880	2,233	42–61 dBA
McNamee et al. (2006)*	Mean daily noise expo-sure level with adjustment for usage of HPDs (L_EP,d_ in dBA; number (N) of years with L_EP,d_> 85 dBA; noise emmission level NIL (NIL=L_EP,d_ +10×log N)	Individual	Experts judgements based on company work histories and noise survey records	Extrapolation	Unexposed, <85 dBA; >1 year exposed to >85 dBA	Total −1402, cases − 717, control − 685	Total −800, cases − 384, control − 416	<85 dBA
Pettersson et al. (2020)	Noise exposure was defined on a job exposure matrix	Group level: a noise exposure category was assigned for each working group in the cohort	Job-exposure matrix	Survey of working conditions carried out by industrial hygienists	≤ 85 dBA; >85 dBA (after re-calculation by authors)	54,480	111,608	≤85 dBA
Song (2013)*	Cumulative noise exposure (dBA-years)	Individual level	Job-exposure matrix	Job-exposure matrix and record linkage	< 85; 85–95; > 95 dBA-years	Cases/controls: 69/347 (85–95 dB); 76/419 (> 95 dB)	Cases/controls: 76/339 (<85 dBA)	<85 dBA-years
Stokholm et al. (2013a)	Mean, full-shift noise exposure levels (L_Aeq_ values in dBA) + cumulative exposure	Individual	Technical device	Measurements	< 70 dB; > 80 dBA for <3, 3–9, 10–19, and ≥20 years; > 80 dBA for <3, 3–9 (used in our analyses), 10–19, and ≥20 years	87,959 men, 15,728 women	20,443 men, 21,060 women	< 70 dB
Stokholm et al. (2013b)	Mean, full-shift noise exposure levels (LAEq values in dBA	Individual	Technical device	Measurements and extrapolation	< 70 dB; > 80 dBA for <3, 3–9, 10–19, and ≥20 years; > 80 dBA for <3, 3–9 (used in our analyses), 10–19, and ≥20 years	496,036	425,763	< 70 dB
Suadicani et al. (2012)	Exposure to noise at a level where it is necessary to raise voice	Individual level	Questionnaire	Self-reported vocal effort	Exposure to “loud” noise for > 1 years	2,998 workers	1,890 workers, noise level 0 years	0 years of exposure to “loud” noise
Tessier-Sherman et al. (2017)	Exposures ever equal or exceed an 8-h time-weighted average	Individual level	Technical device, personal dosimetry measurements	Dosimetry	<82 dBA (referent); 82–84 dBA; 85–87 dBA; >88 dBA (> 82 dBA combined for our analyses)	1,102	950	Occupational exposure to noise <82 dBA
Tong et al. (2017)*	1) The 40-hour time-weighted average (TWA) sound level, in dBA, 2) A cumulative noise exposure (CNE), in dBA x year (dBA-year)	Individual level	Technical device	Cumulative noise exposure (CNE) was determined taking into account noise levels and the years of noise exposure; time-weighted average according the type of work, detention time, and work shift situation (used in our analyses)	<85 dBA; ≥85 dBA time-weighted average (used for our analyses)	461	474	<85 dBA time-weighted average
Virkkunen et al. (2005)	Exposure to continuous noise (used in our analysis), exposure to impulse noise & continuous noise	Individual	Job-exposure matrix	Job-exposure matrix and record linkage	< 80 dBA; 80-85 dBA; >85 dBA dBA	2,893	3,556	< 80 dBA

**Study**	**Outcome assessment**								
Study ID	Definition of outcome	Which International Classification of Diseases (ICD) code was reported for the outcome (if any)?	Method of outcome assessment	Diagnostic assessment method	Specification of outcome	Number of cases with outcome of interest in exposed group	Number of non-cases (i.e. without outcome of interest) in exposed group	Number of cases with outcome of interest in unexposed group	Number of non-cases (i.e. without outcome of interest) in unexposed group
Chang et al. (2013)	Hypertension	None	Questionnaire, Blood pressure measurements	Self-reported diagnosed hypertension or SPB≥ 140 mmHg and/or DBP ≥90 mmHg	Incident hypertension	141	437	44	161
Davies (2002)	Hypertensive heart disease; ischaemic heart disease (IHD); acute myocardial infarction; stroke mortality	Hypertensive diseases (ICD9 401–405.9); IHD (ICD9 411–414.9, 429.2); acute myocardial infarction (ICD 410–410.9); stroke (cerebrovascular disease, ICD9 430–438.9)	Death certificate	Administrative record	Hypertensive heart disease; ischaemic heart disease; acute myocardial infarction; stroke mortality	In the groups > 3 years: hypertensive heart disease (n = 22), IHD (n = 693), acute MI (n = 757), stroke (n = 325)	Unclear	In the reference group < 3 years: hypertensive heart disease (n = 4), IHD (n = 123), acute MI (n = 153), stroke (n = 48)	Unclear
Eriksson et al. (2018b)	Coronary heart disease and stroke	ICD-8, ICD-9, ICD-10. CHD-410–414 (ICD-8, 9), and 120–125 (ICD-10); acute myocardial infarction 410 and 121; stroke 431–438 (ICD −8,9), 161–169 (ICD-10)	Hospital discharge national register	Hospital discharge national register	CHD, stroke, MI	CHD-medium noise 453, high noise 71; Stroke- medium noise 220, high noise 35	CHD - medium noise 2014; high noise −285 Stroke medium noise −2247, high noise −321,	CHD − 480, stroke-262	CHD 2450 Stroke 2668
Girard et al. (2015)*	CVD mortality	ICD-9: 410, ICD-9: 411–414 + 429.2), CI M9 390–405; 415–459 (except 429.2)	Death certificate	Administrative record	Incident CVD mortality	74 (exposed cases)	0 (exposed cases)	87 (unexposed cases)	0 (unexposed cases)
Gopinath et al. (2011)	Angina, acute myocardial infarction, stroke	ICD − 9] codes 410.0, 411.0–8, 412, 414.0–9 and ICD- 10 (121.0–9, 122.0–9, 123.0–8, 124.0–9, 125.0–9, ICD −9: 430.0–438.9 and ICD-10160.0–169.9)	Medical history of participants, Australian National Death Index	Unclear	Prevalence/incidence of angina, acute myocardial infarction, stroke	Angina − 126 13.8%), AMI-98 (10.7%), stroke − 38 (4.1%), all CVD-171 (18.2%)	675	Angina − 168 (9.2%), AMI-115 (6.4%), stroke − 80 (4.4%), all CVD −218 (17.7%)	1496
Huo Yung Kai et al. (2018)*	Hypertension	None	BP was measured using an automatic standard sphygmomanometer (OMRON 705CP)	SBP ≥ 140 mmHg and/or a DBP ≥ 90 mmHg and/or taking a antihypertensive medication	Hypertension	26	99	108	542
Ising et al. (1997)	Myocardial infarction	ICD 410	Hospital discharge record	Hospital discharge record	Myocardial infarction	246	927	149	1221
Kersten and Backe (2015)	Myocardial infarction	None	Computer assisted standardized interview	Physician diagnostic record	Myocardial infarction	166	199	1493	1658
McNamee et al. (2006)*	IHD mortality	ICD-9: 410–414	Death certificate	Administrative record	Incident ICD mortality	717 (exposed cases)	685 (unexposed control)	384 (unexposed cases)	416 (unexposed control)
Pettersson et al. (2020)	Myocardial infarction and stroke	IHD: ICD-8410–412, ICD- 9410–412, and ICD-10I21-I25; Stroke: ICD-8430–438, ICD-9430–438, and ICD-10I60-I69	National Cause of Death Register	Administrative record	Myocardial infarction and stroke	Myocardial infarction: 1,943 Stroke: 534	Myocardial infarction: 52,537 Stroke: 53,946	Myocardial infarction: 4,164 Stroke: 1,116	Myocardial infarction: 107,444 Stroke: 110,492
Song (2013)*	CVD	None	Questionnaire	Self-reported heart disease	Positive response	64 (85–95 dB) /78 (> 95 dB)	331 (85–95 dB) /419 (> 95 dB)	76	339
Stokholm et al. (2013a)	Hypertension	ICD-8 codes, ICD-10 codes, but exact codes uncelar	Data on redeemed anti-hypertensive prescription, hospital discharge	Administrative record	Incidence of hypertension /1000 person-year	Men 6,051 Women 1,603	Men 81,908 Women- 19,457	Men 1,536, Women 2,205	Men 18,907, Women 18,855
Stokholm et al. (2013b)	Stroke	DI61, DI63 D164	Danish National Patient Register	Unclear	Incident stroke	638	Unclear	343	Unclear
Suadicani et al. (2012)	IHD mortality	IHD codes 410–412, ICD (1994) 120–125	Danish National Civil Registry	Physician diagnoses in national registry	IHD mortality	197 deaths due to IHD	2,801	6.4% of 1890 subjects	93.6%
Tessier-Sherman et al. (2017)	Hypertension	ICD9, 401–404	Central data processing vendor for all employees	Administrative datasets	Hypertension	244	1,808	No data	No data
Tong et al. (2017)*	Hypertension	None	Physical examination	Physician diagnostic record	Hypertension	182	279	130	344
Virkkunen et al. (2005)	Coronary heart disease	CHD - codes 410–414 in the ninth revision of the ICD and I20-I25 in the tenth revision	CHD end points were obtained from official Finnish registers	Hospital discharge record	Coronary heart disease	515	2378	509	3047

**Study**	**Adjustments of effect estimates in model prioritized by reviewers**									
Study ID	Adjusted for confounding by: age	Adjusted for confounding by: sex	Adjusted for confounding by: Socioeconomic status (please specify indicator, e.g. level of education)	Other potential confounders adjusted for (please specify)	Adjusted for mediation by: tobacco smoking	Adjusted for mediation by: Alcohol use	Adjusted for mediation by: obesity	Other potential mediators adjusted for	Interactions adjusted for	Adjustment for clustering (if any)
Chang et al. (2013)	Yes	N/A (males only)	Educational level	Body mass index, employment duration, cigarette use, alcohol intake, exercise	Yes	Yes	Yes	No	No	No
Davies (2002)	Yes	N/A (males only)	No	Calendar year and race	No	No	No	No	No	No
Eriksson et al. (2018b)	Yes	N/A (males only)	No	No	No	No	No	No	Interaction between occupational noise and high strain	No
Girard et al. (2015)*	Yes	N/A (males only)	No	No	No	No	No	No	No	No
Gopinath et al. (2011)	Yes	Yes	Occupational prestige	Body mass index, smoking, walking difficulties and self-reported poor health	Yes	No	Yes (stroke incidence model)	Yes	No	No
Huo Yung Kai et al. (2018)*	Yes	Yes	Educational attainment	Body mass index, smoking habits, daily alcohol intake, leisure time physical activity, history of diabetes, history of hypercholesterolemia, treatment for hypertension, working status and initial blood pressure	No	No	No	Yes	No	No
Ising et al. (1997)	Yes	N/A (males only)	Social class, education, marital status, housing area	Body mass index, Social class, Education, Marital status, residential area, shift work, Current smoking	Yes	No	Yes	No	No	No
Kersten and Backe (2015)	Yes (matching variable)	Yes (matching variable)	Current employment status, <12 years at school	Shift work, work >40h per week	No	No	No	No	No	No
McNamee et al. (2006)*	Yes	N/A (males only)	No	Pre-employment measures and duration of employment	No	No	No	No	No	No
Pettersson et al. (2020)	Yes	N/A (males only)	No	Region	No	No	No	No	No	Yes
Song (2013)*	Yes (matching variable)	Yes (matching variable)	Education, family income	Smoking, body mass index, drinking, smoking, physical activity, hypertension	Yes	Yes	Yes	Yes	No	No
Stokholm et al. (2013a)	Yes	Yes	Five categories, blue/white collar	Calendar year, employment status	No	No	No	No	Interaction between sex and occupation	Yes
Stokholm et al. (2013b)	Yes	Yes	Socioeconomic status	Calendar year, employment status	No	No	No	No	No	No
Suadicani et al. (2012)	Yes	N/A (males only)	Low social class	Physical activity, cumulative tobacco consumption, alcohol intake	Yes	Yes	Yes	No	Age + lifestyle and social class, age + clinical factors, age + all potential confounders	No
Tessier-Sherman et al. (2017)	Yes	N/A (males only)	Economic status, job category, annual wages	Body mass index, smoking, hearing acuity	Yes	No	Yes	Yes	No	Yes
Tong et al. (2017)*	No	N/A (males only)	No	Body mass index, low density lipoprotein cholesterol, hypertension family history, A1166C gene	Yes	No	No	Yes	No	No
Virkkunen et al. (2005)	Yes	N/A (males only)	No	Systolic blood pressure	No	No	No	Yes	No	No

**Study**	**Prioritized model**			**Estimate of effect of exposure on outcome**	
Study ID	Are two or more alternative models reported?	Which of the alternative models was prioritized/selected for use in the review and/or meta-analysis?	Reason for prioritization/selection	Treatment effect measure type	Was an exposure–response (or dose–response) analysis conducted?
Chang et al. (2013)	Yes	Relationships between noise exposure and hypertension in total	N/A	Hazard ratio	No
Davies (2002)	Yes	The model yielding RR of different cardiovascular outcomes in those exposed to > 85 dBA for >3 years vs. exposed to >85 dBA for <3 years	This duration of exposure was most biologically plausible, as exposed for <3 years would be unlikely to cause cardiovascular disease	Relative risk	Trend per increasing duration of exposure (not of interest for pooling)
Eriksson et al. (2018b)	Yes – age-adjusted and fully-adjusted model (body mass index, diabetes, hypertension, smoking, cholesterol)	Hazard ratio adjusted for age only	Overadjustment for potential mediators in the fully-adjusted model	Hazard ratio	No
Girard et al. (2015)*	Yes – models for duration of noise exposure and crude 2x2 table	Raw data in descriptive	The duration of exposure categories are not comparable to the exposure categories in other studies	Calculated relative risk	No
Gopinath et al. (2011)	Yes – incidence and mortality	Only the mortality model, because for the incidence model, the only significant effect was selectively reported, and it was based on only 4 cases with stroke	N/A	Hazard ratio and Odds ratio	Trend per increasing duration of exposure (not of interest for pooling)
Huo Yung Kai et al. (2018)*	Yes, crude and adjusted models (age, gender, body mass index, smoking, alcohol, physical activity, diabetes, hypercholesterolemia, employment, educational attainment)	Unadjusted model (calculated from raw data) due to gross adjustment for mediators in the adjusted model; Moreover, adjusted estimate was reported only in a Fig. with poor resolution	The adjusted models revealed that most of these associations were explained by individual cardiovascular factors, except for the negative effect of high job strain and positive effect of job recognition which had an independent role	Odds ratio reported, but we calculated relative risk from raw data	No
Ising et al. (1997)	Yes – crude and adjusted models	Model adjusted for smoking, body mass index, age, social class, education, marital status, shift work, housing area	Control for confounding factors with acceptable adjustment for potential mediators	Odds ratio	No
Kersten and Backe (2015)	Yes – models using all occupational groups and stratified by occupational group	The model using all occupational groups combined (for men and women)	Insufficient number of cases in the stratified models	Odds ratio	No
McNamee et al. (2006)*	Yes – crude and two adjusted; data from both sampling sites vs. data from one site	Adjusted model taking into account both sites	Control for confounding factors and acceptable adjustments for potential mediators; Moreover, the estimates do not differ between crude and adjusted models	Odds ratio	Yes
Pettersson et al. (2020)	Yes – re-calculated upon request	Relative risk adjusted for age and region	Parsimony	Relative risk	No
Song (2013)*	Yes – crude and adjusted models	Multivariate logistic regression	Control for confounding factors and acceptable adjustments for potential mediators	Odds ratio	No
Stokholm et al. (2013a)	Yes – crude and adjusted models	Adjusted model	Control for confounding factors	Relative risk	No
Stokholm et al. (2013b)	Yes – crude and adjusted models	Adjusted model	Control for confounding factors	Hazard ratio	Trend RR by 1-unit dBA-year increase (not of interest for pooling)
Suadicani et al. (2012)	Yes – four models	Age + lifestyle and social class-adjusted model	This model seems the best compromise between parsimony and controlling for confounders	Hazard ratio	No
Tessier-Sherman et al. (2017)	Yes – crude and adjusted models	Model adjusted for age, body mass index, smoking	Control for confounding factors and acceptable adjustments for potential mediators	Relative risk	Yes
Tong et al. (2017)*	Yes – models for time-weighted average and cumulative noise exposure	Time-weighted average model	Allows comparison with the other studies that used this noise metric	Odds ratio	No
Virkkunen et al. (2005)	Yes – different follow-up models and estimates for continuous and impulse noise	The longest follow-up model and continuous noise	The other follow-ups yield similar effect estimates; relatively few workers are exposed to impulse noise	Relative risk	Trend per increasing level of exposure (could not be pooled)

*The study provided deprioritized evidence and was not included in the main meta-analysis due to it being a single case-control study in the respective model (Girard et al., 2015, McNamee et al., 2006, Tong et al., 2017), unadjusted estimate extracted (Huo Yung Kai et al., 2018) or incomparable noise metric (Song, 2013).

#### 4.2.1 Study type

Most studies were cohort studies (11 studies), followed by case-control studies (six studies) (Table 4 Part I). We extracted six RRs (one calculated from raw data), seven ORs and five HRs. (Gopinath et al., 2011) reported both a HR and an OR. Most studies adjusted for at least one of our pre-specified confounders. Note that from some studies only crude estimates could be extracted; for example, Huo Yung Kai et al. (2018) did adjust for all predefined confounders (including potential mediators), but the results from that model were only reported in one of the figures in that paper and could not be digitized accurately due to poor resolution. Among the potential mediators, the most commonly adjusted for were body mass index, tobacco, cholesterol levels, alcohol consumption (see Table 4 Part IV).

#### 4.2.2 Population studied

The studies included about 534,688 workers (>93% males). The most commonly studied age groups were those between 20 and 65 years. By WHO region, most studies examined populations in the European region (ten studies from six countries), followed by populations in the Americas (four studies from two countries) and populations in the Western Pacific (three studies from three countries). More than one study came from Denmark and Canada (three studies each), Sweden and Germany (two studies each). The industrial sectors most commonly studied were manufacturing of wood (one study), machineries (one study) and metals (five studies), followed by construction, agriculture and mining (two studies each). The workers in most studies were craft and related trades workers (eight studies), followed by technicians and associate professionals (one study). The other studies did not provide quantitative breakdowns of participants by industrial sectors and occupation, but they did appear to cover several industrial sectors and occupations (Table 4 Part I).

#### 4.2.3 Exposure studied

Most studies measured occupational exposure to noise with dosimetry, sound level meter or official company records. Some studies relied on validated questions on self-reported noise exposure (four studies), and three studies used a job-exposure matrix (JEM) (Table 4 Part II).

#### 4.2.4 Comparator studied

The comparator in most studies was <85 dBA. In some studies, the comparator was exposure to ≥85 dBA for <3 years (Davies et al., 2005, Suadicani et al., 2012). We assumed that exposure for a short period of time (<3 years) was not expected to have caused CVD and therefore could serve as a reasonable reference group. Other studies used a lower cut-off level, <80 dBA (Virkkunen et al., 2005), <70/75 dBA (Eriksson et al., 2018b, Ising et al., 1997, Stokholm et al., 2013a), or even <61 dBA (Kersten and Backe, 2015), which was still below the theoretical minimum risk exposure level of < 85 dBA. Girard et al. (2015) used an exposure cut-off level of 90 dBA (Table 4 Part II).

#### 4.2.5 Outcomes studied

All studies reported evidence on the outcome prevalence of, incidence of and mortality from CVD. Of these, five studies (of which two cohort studies) defined the outcome as IHD incidence, six studies (of which four cohort studies) as IHD mortality, three studies (of which two cohort studies) as stroke incidence, three cohort studies as stroke mortality, and five studies (of which four cohort studies) as hypertension incidence. Song (2013) used the unspecific self-reported diagnosis with “heart disease”, which we assumed referred to IHD. Outcome assessment was objectively measured (e.g., by administrative health records) in the majority of studies (Table 4 Part III).

### 4.3 Risk of bias at individual study level

The detailed justification for each rating for each domain by included study is presented in Appendix 5 in the Supplementary data.

#### 4.3.1 Acquired IHD (IHD incidence)

**Fig. 3:**
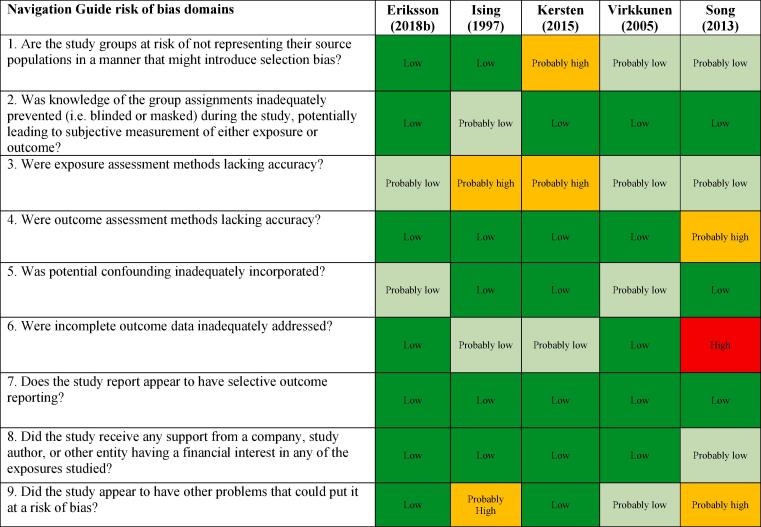
Summary of risk of bias across studies, Acquired IHD (IHD incidence).

The ratings in different risk of bias domains for all five included studies on IHD incidence are presented in Fig. 3.

##### 4.3.1.1 Selection bias

We assessed risk of bias in this domain based on whether the groups being compared were the same in all relevant ways (or as close to this as possible) apart from the exposure and representative of the source population. Of the five included studies, the risk of selection bias was rated to be “probably high” for one study due to a lack of clear identification of the source population; furthermore, a hospitalized sample is at risk of not representing the general population when hospital controls are used. Song, 2013, Virkkunen et al., 2005 were rated to have a “probably low” risk of bias.

##### 4.3.1.2 Performance bias

For the included studies, blinding of study participants and study personnel to assignments of study participants to occupational exposure to noise and to study participants’ characteristics was usually not reported in the study’s record or records. However, we judged that lack of blinding was unlikely to have influenced the outcome and exposure measures in record-linkage studies. Therefore, we rated the risk for all studies as “low”. Only the Ising et al. (1997) study was rated as having a “probably low” risk (Fig. 3).

##### 4.3.1.3 Detection bias (exposure assessment)

We rated three studies as carrying a “probably low” risk of detection bias in the exposure assessment. Eriksson et al., 2018b, Virkkunen et al., 2005, Song, 2013 used a JEM for noise based on measurements, which is a standard exposure assessment approach in the field, although it is an indirect measure of exposure with limited accuracy on the individual-level. The other two studies received a “probably high” rating. Kersten and Backe (2015) validated their noise estimates in a subsample where it correlated with measured noise levels, and accounted for long-term exposure and hearing protector use; however, no reliable information was available on the specific noise measurement equipment used or calibration procedures and accuracy at the individual-level could be low. Ising et al. (1997) used a subjective exposure scale based on sound intensity of common noise sources, verifying the correlation between subjective and objective noise levels in a small subsample of 80 subjects. The authors consider that the retrospective assessment of the exposure level in this study could have been influenced by the experience of myocardial infarction, leading to a systematic over-estimation of noise by myocardial infarction survivors.

##### 4.3.1.4 Detection bias (outcome assessment)

In four of the studies, outcome assessment was objective and the risk of bias rated as “low”. However, Song (2013) only had information on self-reported “heart disease”, which is prone to reporting bias and does not match specific CVD taxonomy. Thus, for this study we rated the risk of bias as “probably high” (Fig. 3).

##### 4.3.1.5 Confounding

This bias was rated as “probably low” for two studies because they accounted for two out of three important confounders but did not adjust for socioeconomic position (Eriksson et al., 2018b, Virkkunen et al., 2005). The other studies were judged to be at “low” risk of bias (Fig. 3).

##### 4.3.1.6 Selection bias (incomplete outcome data)

This bias was rated as “low” for two studies, “probably low” for two, and “high” for Song (2013). Ising et al., 1997, Ising et al., 1997, Kersten and Backe, 2015 drew cases from major Berlin hospitals but some smaller hospitals were not included in the sampling. In the Song (2013) study, the number of those with unknown cardiovascular disease status in the original sample was greater than the total analysis sample in the study, which could have induced biologically relevant bias in the effect estimate (Fig. 3).

##### 4.3.1.7 Reporting bias

We judged all included studies to be at “low” risk of reporting bias. In case-control studies with a predefined outcome, this bias was of no concern. In the other studies, the outcomes were reported as they had been pre-specified in the protocol and in the methods section (Fig. 3).

##### 4.3.1.8 Conflict of interest

This bias was rated as “probably low” for one study because there was no conflict of interest statement or a disclosure of competing interests (Song, 2013). Nevertheless, the study was reported in a Master of Science thesis and it is unlikely that conflict of interest existed. The other studies were rated as having a “low” risk of bias because we identified no conflict of interest or funding sources that could have influenced their conduct or reporting (Eriksson et al., 2018b, Ising et al., 1997, Kersten and Backe, 2015, Virkkunen et al., 2005) (Fig. 3).

#### 4.3.2 Other risk of bias

Two studies received a “probably high” rating (Ising et al., 1997, Virkkunen et al., 2005) because they adjusted for multiple potential mediators. One other study also adjusted for one mediator (systolic blood pressure) (Ising et al., 1997, Song, 2013, Virkkunen et al., 2005), but that did not seem to reduce the effect size. The remaining studies were judged to be at “low” risk of bias in this domain (Fig. 3).

#### 4.3.3 Died from IHD (IHD mortality)

**Fig. 4:**
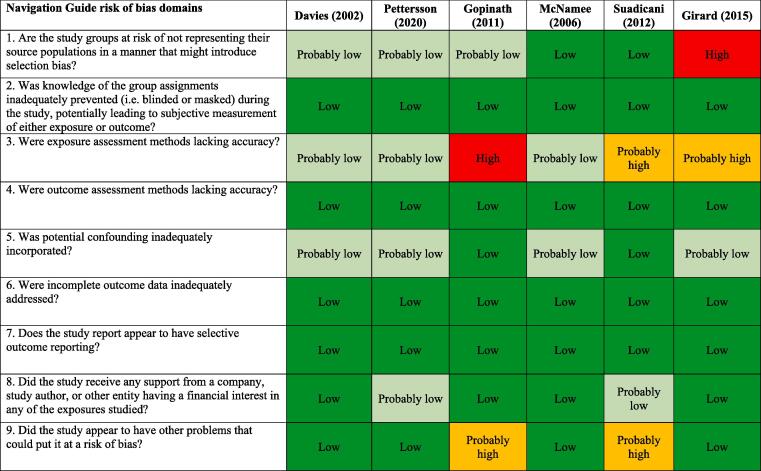
Summary of risk of bias across studies, Died from IHD (IHD mortality).

The ratings in different risk of bias domains for all six included studies on IHD mortality are presented in Fig. 4.

##### 4.3.3.1 Selection bias

This bias was rated as “probably low” for three studies because participation in the study was hampered by high attrition rates. However, the descriptions of the source population, inclusion/exclusion criteria, recruitment and enrolment procedures and follow up studies were sufficiently detailed and there was no evidence to suggest inconsistencies across groups (Davies, 2002, Girard et al., 2015, Gopinath et al., 2011). Girard et al. (2015) received a “high” risk of bias rating because there was a drastic reduction in sample size from 8910 in the source population to 644 in the current study, which may have introduced an unknown degree of bias related to the exposure, as many workers were excluded based on audiometric results and hearing loss status (Fig. 4). The other two studies were considered at “low” risk of bias.

##### 4.3.3.2 Performance bias

This bias was rated as “low” for all studies because they were based on secondary analysis of data collected for other purposes with no access to information that could identify subjects; therefore, noise assessment was independent of group status. In the Girard et al. (2015) study, for example, lack of blinding was also unlikely to have introduced bias because the original sampling and audiometric testing were carried out to study the effect of noise on hearing loss not CVD (Fig. 4).

##### 4.3.3.3 Detection bias (exposure assessment)

The Davies (2002) study received a “probably low” rating because although it used a valid combination of measurements, including personal noise dosimetry, interviews, hygienists' assessment and modelling, non-differential exposure misclassification could have been at play. Two other studies (McNamee et al., 2006, Pettersson et al., 2020) received a “probably low” rating because they used a JEM for noise based on measurements, which is a standard exposure assessment approach in the field, although it is an indirect measure of exposure with limited accuracy on the individual-level. The Girard et al. (2015) study was judged to be at “probably high” risk of bias because it relied on a single workplace measurement of noise exposure and, as the authors noted, that noise level was not representative of the worker’s long-term exposure. Suadicani et al. (2012) was also rated as “probably high” because it relied on self-reported measure, which although a proxy for noise exposure, may introduce differential exposure misclassification and between-worker variation due to individual differences. We rated this bias as “high” for one study because it used a questionnaire including a dichotomized question on having ever been exposed to noise at the workplace and severity of noise to assess exposure. This question was not completely in line with the standard wording of validated questions using vocal effort to overcome ambient noise as a proxy for noise exposure (Gopinath et al., 2011) (Fig. 4).

##### 4.3.3.4 Detection bias (outcome assessment)

We rated the risk of detection bias for all these studies as “low”, because studies used official/objective medical records and outcome assessment was based on standardized medical information (Fig. 4).

##### 4.3.3.5 Confounding

This bias was rated as “probably low” in four studies Three of them accounted for age and sex only (Davies, 2002, McNamee et al., 2006, Pettersson et al., 2020). Girard et al. (2015) accounted for all three important confounders by matching on follow-up duration and industrial sector (proxies for age and socioeconomic position) and including only male participants; however, the assumption that age and socioeconomic position were accounted for this way was tentative (Fig. 4).

##### 4.3.3.6 Selection bias (incomplete outcome data)

This bias was rated as “low” for all studies. There was no incomplete outcome data suspected as the data sources were medical records/databases (Fig. 4).

##### 4.3.3.7 Reporting bias

We judged risk of reporting bias as “low” in all included studies. The outcomes were reported in the included study record as they had been pre-specified in the protocol and in the abstracts and methods sections in the study record (Fig. 4).

##### 4.3.3.8 Conflict of interest

This bias was rated as “low” for four studies because no conflict of interest was suspected based on authors’ affiliations and funding sources. However, for two studies (Pettersson et al., 2020, Suadicani et al., 2012) it was rated as “probably low” because one was funded by an insurance company, which could have interest in the outcomes of the study, and the other one was funded by several foundations even though a statement of no conflict of interest was provided. Still, the authors were affiliated with public research institutions and health universities, which makes competing interests unlikely (Fig. 4).

##### 4.3.3.9 Other risk of bias

Two studies had overadjusted their models for several potential mediators, which could have produced conservative findings, so they received a “probably high” rating (Gopinath et al., 2011, Suadicani et al., 2012) (Fig. 4).

#### 4.3.4 Summary of risk of bias across studies, Acquired stroke (stroke incidence)

**Fig. 5:**
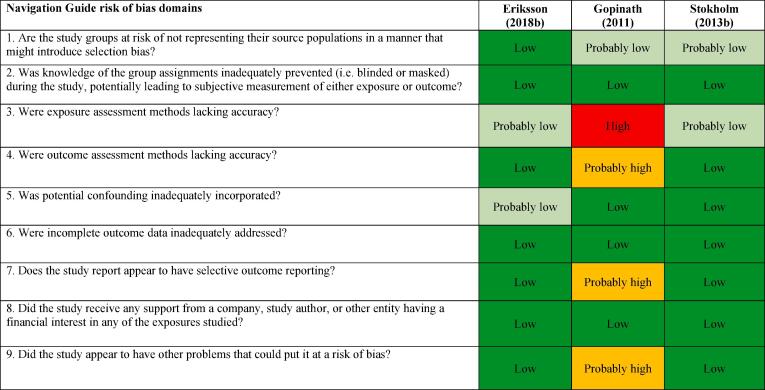
Summary of risk of bias across studies, Acquired stroke (stroke incidence).

The ratings in different risk of bias domains for the three included studies on stroke morbidity are presented in Fig. 5.

##### 4.3.4.1 Selection bias

Of the three included studies, the risk of selection bias was rated to be “probably low” for two studies. In the study by Gopinath et al. (2011), there was progressive reduction in the response rate across the survey cycles, but we did not suspect that inclusion/exclusion criteria, recruitment, and participation and follow-up rates differed systematically between cases and controls. Stokholm et al. (2013b) analysed half of the original size of the source population, however, we had no serious concerns that inclusion/exclusion criteria, recruitment and participation rates differed systematically between cases and controls. Eriksson et al. (2018b) received a “low” risk of bias rating (Fig. 5).

##### 4.3.4.2 Performance bias

This bias was rated as “low” for all three studies because there was no direct access to the study population as they were all record-linkage studies. We judged that lack of blinding was unlikely to have influenced the outcome and exposure measures (Fig. 5).

##### 4.3.4.3 Detection bias (exposure assessment)

This bias was rated as “high” for one study because the exposure assessment was based on a questionnaire including a dichotomized question on having ever been exposed to noise at the workplace and severity of noise exposure, which was not completely in line with the standard wording of validated questions using vocal effort to overcome ambient noise as a proxy for noise exposure (Gopinath et al., 2011). The other two studies received a “probably low” rating because they used a JEM for noise based on measurements, which is a standard exposure assessment approach in the field, although it is an indirect measure of exposure with limited accuracy on the individual-level (Eriksson et al., 2018b, Stokholm et al., 2013b) (Fig. 5).

##### 4.3.4.4 Detection bias (outcome assessment)

This bias was rated as “probably high” for one study because stroke diagnosis was determined through an interviewer-administered questionnaire (Gopinath et al., 2011) (Fig. 5). The other two studies received a “low” risk of bias rating because the outcome was assessed based on official/objective medical records and medical information from national diagnosis or patient registers.

##### 4.3.4.5 Confounding

The bias was rated as “probably low” in one study because it only adjusted for two out of three important confounders (Tier I) but did not adjust not for socioeconomic status (Eriksson et al., 2018b). This bias was rated as “low” for the other two studies (Fig. 5).

##### 4.3.4.6 Selection bias (incomplete outcome data)

This bias was rated as “low” for all studies because no substantive bias was suspected (Fig. 5).

##### 4.3.4.7 Reporting bias

In the study by Gopinath et al. (2011), this bias was rated as “probably high” because the authors reported the estimate for “the only significant association observed with stroke among those exposed to severe level of noise exposure for less than 1–5 years”. For the other studies, the reporting was consistent with the pre-specified outcomes and they were judged to be at “low” risk of bias. (Fig. 5).

##### 4.3.4.8 Conflict of interest

This bias was rated as “low” for all studies as inspection of funding sources and authors’ affiliations did not reveal evidence of conflict of interest (Fig. 5).

##### 4.3.4.9 Other risk of bias

One study had overadjusted its model for several potential mediators and received a “probably high” rating (Gopinath et al., 2011) (Fig. 5).

#### 4.3.5 Summary of risk of bias across studies, Died from stroke (stroke mortality)

**Fig. 6:**
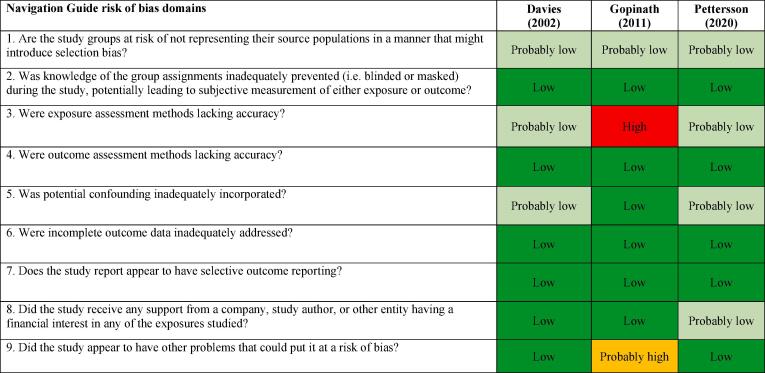
Summary of risk of bias across studies, Died from stroke (stroke mortality).

The ratings in different risk of bias domains for the three included studies on stroke mortality are presented in Fig. 6.

##### 4.3.5.1 Selection bias

This bias was rated as “probably low” for all studies because participation in the study may have been hampered by high attrition rates. However, the descriptions of the source population, inclusion/exclusion criteria, recruitment and enrolment procedures and follow up studies were sufficiently detailed and there was no evidence to suggest inconsistencies across groups (Davies, 2002, Girard et al., 2015, Gopinath et al., 2011).

(Fig. 6).

##### 4.3.5.2 Performance bias

This bias was rated as “low” for all three studies because there was no direct access to the study population as these are all record-linkage studies; we judged that lack of blinding is unlikely to influence the outcome and exposure measures in record-linkage studies. (Fig. 6).

##### 4.3.5.3 Detection bias (exposure assessment)

This bias was rated as “high” for one study because the exposure assessment was based on a question on having ever been exposed to noise at the workplace and severity of noise exposure, which was not completely in line with the standard wording of validated questions using vocal effort to overcome ambient noise as a proxy for noise exposure (Gopinath et al., 2011). For the other two studies, it was rated as “probably low” because they used a JEM for noise based on measurements, which is a standard exposure assessment approach in the field, although it is an indirect measure of exposure with limited accuracy on the individual-level (Davies, 2002, Pettersson et al., 2020) (Fig. 6).

##### 4.3.5.4 Detection bias (outcome assessment)

This bias was rated “low” for all three studies, because the outcome was assessed based on official/objective medical records and medical information from national diagnosis or patient registers (Fig. 6).

##### 4.3.5.5 Confounding

This bias was rated as “probably low” in two studies (Davies, 2002, Gopinath et al., 2011, Pettersson et al., 2020) because they accounted for age and sex but did not adjust for socioeconomic status. The other study accounted for all three important confounders (Gopinath et al., 2011) (Fig. 6).

##### 4.3.5.6 Selection bias (incomplete outcome data)

All studies were judged to be at “low” risk of bias because there was no incomplete outcome data suspected as the data source were through medical records/databases (Fig. 6).

##### 4.3.5.7 Reporting bias

We judged risk of reporting bias as “low” in all included studies. The outcomes were reported in the included study record as they had been pre-specified in the protocol and as they had been reported in the abstracts and methods sections in the study record (Fig. 6).

##### 4.3.5.8 Conflict of interest

We did not find any evidence of such a bias in two of the included studies and therefore judged them to have a “low” risk of bias (Davies, 2002, Gopinath et al., 2011). Pettersson et al. (2020) was judged to be at “probably low” risk of bias because it was funded by an insurance company, which could have interest in the outcomes of the study. Still, the authors were affiliated with a research institution and reported no conflict of interest (Fig. 6).

##### 4.3.5.9 Other risk of bias

One study received a “probably high” rating because it overadjusted for several potential mediators (Davies, 2002, Gopinath et al., 2011, Pettersson et al., 2020) (Fig. 6).

#### 4.3.6 Summary of risk of bias across studies, Acquired hypertension (hypertension incidence)

**Fig. 7:**
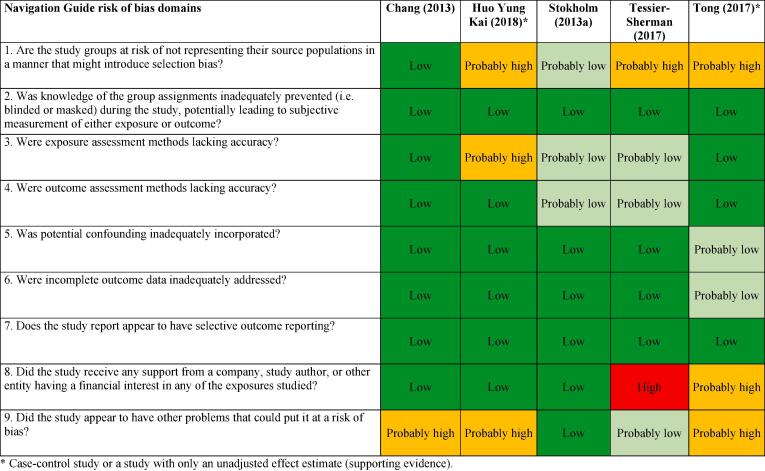
Summary of risk of bias across studies, Acquired hypertension (hypertension incidence). * Case-control study or a study with only an unadjusted effect estimate (supporting evidence).

The ratings in different risk of bias domains for the five included studies on hypertension are presented in Fig. 7.

##### 4.3.6.1 Selection bias

Of the five included studies, the risk of selection bias was rated to be “low” for one study (Chang et al., 2013) and “probably low” for Stokholm et al. (2013a). It was rated as “probably high” in the three remaining studies because of high attrition rate and systematic differences between included and dropout participants (Huo Yung Kai et al., 2018) or potential differences between included workers and all employees in the sampling company (Tessier-Sherman et al., 2017, Tong et al., 2017) (Fig. 7).

##### 4.3.6.2 Performance bias

We did not find any evidence of performance bias and therefore judged all included studies to have a “low” risk of bias in this domain (Fig. 7).

##### 4.3.6.3 Detection bias (exposure assessment)

The risk of selection bias was rated to be “probably high” for one study. In the study by (Huo Yung Kai et al., 2018), the exposure was measured with a question on individual perception of noise level at the work place, which may introduce differential exposure misclassification. In Tong et al. (2017) the objective exposure assessment method was not described in detail and there were no personal measurements with a dosimeter. One study received a “probably low” rating because the exposure assessment method was objective and described in detail, personal measurements were collected for each job title to construct a database, and measurements followed Occupational Safety and Health Administration (OSHA) protocol. Still, accuracy at the individual-level could be limited (Tessier-Sherman et al., 2017). Another study also received a “probably low” rating because it used a JEM for noise based on measurements, which is a standard exposure assessment approach in the field, although it is an indirect measure of exposure with limited accuracy on the individual-level (Stokholm et al., 2013a). The other two studies were found to be at “low” risk of bias because they used detailed individual-level measurements (Fig. 7).

##### 4.3.6.4 Detection bias (outcome assessment)

This bias was rated at “low” risk for three studies, where we did not find any evidence of outcome assessment bias (Chang et al., 2013, Huo Yung Kai et al., 2018, Tong et al., 2017). In Tessier-Sherman et al., 2017, Stokholm et al., 2013a this bias domain was rated as “probably low” because in these studies the outcome assessment was based on medical records and, unlike IHD and stroke, hypertension may go undetected, so the real prevalence may be underestimated (Fig. 7).

##### 4.3.6.5 Confounding

This bias was rated as “low” in four studies that adjusted for all the three important confounders (Tier I). One study was rated as “probably low” as it accounted for two out of three important confounders (Tier I) by supporting same mean age across cases and controls and including only male participants (Tong et al., 2017) (Fig. 7).

##### 4.3.6.6 Selection bias (incomplete outcome data)

This bias was rated as “probably low” only for one study owing to a lack of information on missing data and the number of workers who did not participate in the occupational physical examination. Still, we judge that this percentage was not likely to be high as the company carried out this official medical screening (Tong et al., 2017). The other four studies received a “low” rating (Fig. 7).

##### 4.3.6.7 Reporting bias

We judged the risk of reporting bias as “low” in all included studies. The outcomes were reported in the included study record as they had been pre-specified in the protocol and as they had been reported in the abstracts and methods sections in the study record (Fig. 7).

##### 4.3.6.8 Conflict of interest

One study received a “high” risk of bias rating because it was funded by grants from institutions employing some of the authors and from the company where the study was conducted, which partly covered the compensation of some of the authors through a contractual agreement (Tessier-Sherman et al., 2017). Another study received a “probably high” rating because one of the authors was affiliated with the Tangshan Iron and Steel Group Co., Ltd plant (Tong et al., 2017) (Fig. 7).

##### 4.3.6.9 Other risk of bias

In one study, which received a “probably low” rating, the effect estimate of interest was adjusted for potential mediators. However, bivariate and adjusted models did not indicate major impact of those mediators (Tessier-Sherman et al., 2017). Three studies received a “probably high” rating because of adjusting for potential mediators meaningfully affected the effect estimate (Chang et al., 2013) (Fig. 7).

### 4.4 Synthesis of results

#### 4.4.1 Acquired IHD (IHD incidence)

A total of five studies (two cohort and three case-control) with a total of 19,740 participants from two WHO regions reported six estimates of the effect of occupational exposure to noise, compared with no occupational exposure to noise, on the risk of acquiring IHD (IHD incidence). We synthesised evidence from different study designs separately (as per protocol). Evidence from cohort studies was synthesised and treated as “prioritized evidence”; evidence from case-control studies was separately synthesised and treated as “supportive evidence”.

**Fig. 8:**
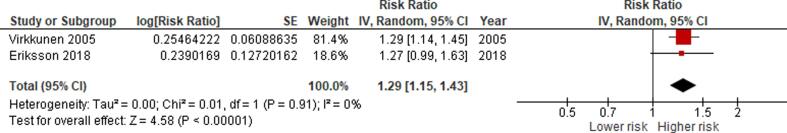
Main meta-analysis of prioritized evidence (cohort studies), Outcome: Acquired IHD (IHD incidence), Comparison: Exposed to ≥85 dBA compared with exposed to <85 dBA.

We considered the two cohort studies (Eriksson et al., 2018b, Virkkunen et al., 2005) to be sufficiently homogenous to be combined in a quantitative meta-analysis. Based on these (Eriksson et al., 2018b, Virkkunen et al., 2005), workers exposed to ≥85 dBA were found to have a 29% higher risk of acquiring IHD, when compared with workers exposed to <85 dBA (RR = 1.29, 95% CI 1.15–1.43, 2 studies, about 11,758 participants, I^2^ = 0%; Fig. 8). With just two estimates, it was not feasible to conduct publication bias tests or leave-one-out meta-analysis. In sensitivity analyses, using risk estimates for the alternative 18-year follow-ups reported in Virkkunen et al. (2005), fixed effects models, and IVhet models yielded comparable results.

**Fig. 9:**
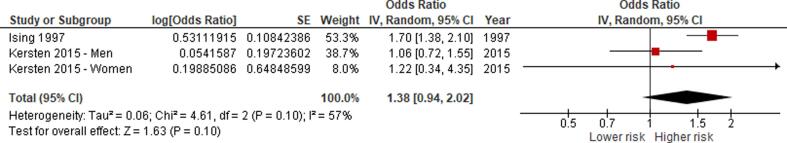
Additional meta-analysis of supportive evidence (case-control studies), Outcome: Acquired IHD (IHD incidence), Comparison: Exposed to ≥85 dBA compared with exposed to <85 dBA.

Three case control studies provided supporting evidence. Of these three studies, we judged two studies (Ising et al., 1997, Kersten and Backe, 2015) supplying three effect estimates to be sufficiently clinically homogenous to be combined in a meta-analysis. The pooled effect estimate from this meta-analysis had an estimate that suggested a 38% increased odds of IHD among those occupationally exposed to noise, compared to those not occupationally exposed to noise (OR 1.38, 95% CI 0.94–2.02, two studies, three estimates, 6656 participants, I^2^ 57%; Fig. 9). This body of evidence from case-control studies supported the results of the main analysis. We excluded the third case-control study (Song, 2013) from the meta-analysis because we judged its noise metric (cumulative noise exposure) to be too different from that used in the other case-control studies (equivalent sound level). It reported that exposure to 85–95 dBA-year and >95 dBA-year was not associated with incidence of “heart disease” (OR 0.87, 95% CI 0.61–1.26 and OR 0.77, 95% CI 0.53–1.14, respectively).

#### 4.4.2 Died from IHD (IHD mortality)

A total of six studies with a total of about 199,570 participants from three WHO regions reported estimates of the effect of occupational exposure to noise, compared with no occupational exposure to noise, on the risk of dying from IHD. We again prioritized the evidence from cohort studies (prioritized evidence) over that from case-control studies (supportive evidence).

**Fig. 10:**
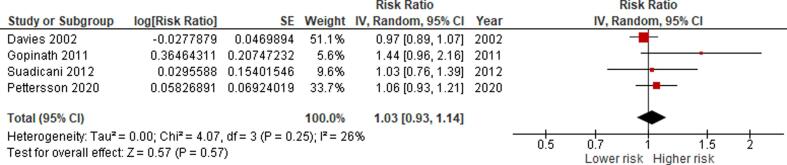
Main meta-analysis of prioritized evidence (cohort studies), Outcome: Died IHD (IHD mortality), Comparison: Exposed to ≥85 dBA compared with exposed to <85 dBA.

The four cohort studies were clinically homogenous enough to be combined in a quantitative meta-analysis (Davies, 2002, Gopinath et al., 2011, Pettersson et al., 2020, Suadicani et al., 2012). Girard et al. (2015) was a case-control study and used a higher exposure cut-off of 90 dBA, therefore it was not pooled with the other case-control study by McNamee et al. (2006). The prioritized pooled effect estimate indicated a very small increased risk (RR = 1.03, 95% CI 0.93 to 1.14, four studies, about 198,926 participants, I^2^ 26%; Fig. 10). Asymmetry in the Doi plot and large LFK index of 3.14 suggested possible publication bias. In leave-one-out meta-analysis, the overall results remained similar. Using alternative estimates from Davies (2002) (10–20 years or for >30 years instead of 3–10 years of exposure), produced roughly the same results as in the main model. If the estimate adjusted only for age from Suadicani et al. (2012) was used, there was no substantive difference from the main model. The fixed effects and IVhet estimators produced virtually the same results.

The two case-control studies, Girard et al., 2015, McNamee et al., 2006, were too clinically heterogenous to be combined in a quantitative meta-analysis – while the McNamee et al. (2006) used the standard comparator, Girard et al. (2015) used a higher exposure cut-off of 90 dBA. Girard et al. (2015) reported a point estimate of 0.86, with the 95% CI crossing the 1.00 (RR 0.86, 95% CI 0.66 to 1.13). McNamee et al. (2006) reported an OR of a 1.13 (95% CI 0.92, 1.39) among exposed workers compared with unexposed workers. We judged this evidence from supporting studies to be similar to that presented in the main analysis.

#### 4.4.3 Acquired stroke (stroke incidence)

**Fig. 11:**
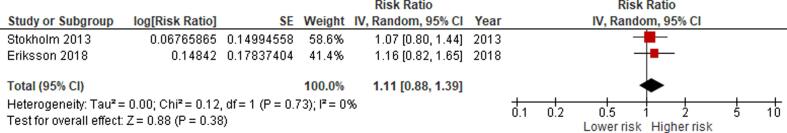
Main meta-analysis of prioritized evidence (cohort studies), Outcome: Acquired stroke (stroke incidence), Comparison: Exposed to ≥85 dBA compared with exposed to <85 dBA.

Three cohort studies with a total of about 171,952 participants from three WHO regions reported estimates of the effect of occupational exposure to noise on the risk of stroke incidence, compared with no occupational exposure to noise. Of these, two studies were sufficiently homogenous (Eriksson et al., 2018b, Stokholm et al., 2013b) to be pooled in a meta-analysis. Workers exposed to ≥85 dBA had a non-significantly higher risk of 11% of acquiring stroke (RR = 1.11, 95% CI: 0.88–1.39, two studies, about 170,000 participants, I^2^ = 0%; Fig. 11). No publication bias and leave-one-out tests were performed. If we used the estimates for 10–19 and ≥20 years of exposure from Stokholm et al. (2013b), the effect was closer to being significant, but the 95% CI remained wide. The results were unchanged using the fixed effects and IVhet estimators.

The third cohort study by Gopinath et al. (2011) reported OR = 3.44 (95% CI 1.11–10.63) for incident stroke among workers exposed to “severe workplace noise” for less than 1–5 years versus no exposure. This cohort study was excluded from the meta-analysis because we believed it to be selectively reported; the model possibly suffered from sparse data bias with only four cases of stroke; and in the higher exposure category no increased risk was observed which we considered biologically implausible or an indication of survivor effect. While this study reported a much higher and statistically significant effect than the evidence in our main analysis, we considered this to be explained by the study’s limitations described above.

#### 4.4.4 Died from stroke (stroke mortality)

**Fig. 12:**
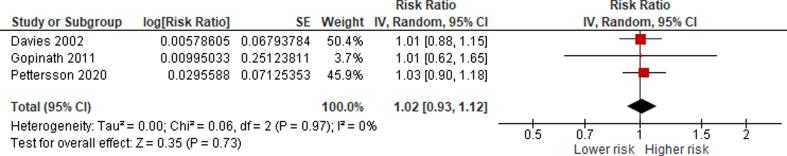
Main meta-analysis of prioritized evidence (cohort studies), Outcome: Died from stroke (stroke mortality), Comparison: Exposed to ≥85 dBA compared with exposed to <85 dBA.

Three cohort studies with 195,539 participants from two WHO region reported estimates of the effect of occupational exposure to noise on the risk of dying from stroke when working exposed to ≥85 dBA, compared with <85 dBA. These studies were sufficiently similar to be combined in one meta-analysis. The prioritized pooled effect estimate from this meta-analysis was close to 1.00 and the 95% CI included 1.00 (RR = 1.02, 95% CI: 0.93–1.12, I^2^ = 0%; Fig. 12). The pooled effect remained robust to exclusion of each study one-at-a-time or using alternative meta-analysis estimators.

#### 4.4.5 Acquired hypertension (hypertension incidence)

**Fig. 13:**
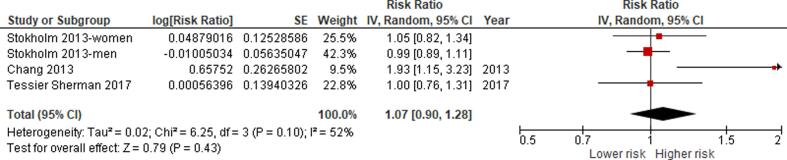
Main meta-analysis of prioritized evidence (cohort studies), Outcome: Acquired hypertension (hypertension incidence), Comparison: Exposed to ≥85 dBA compared with exposed to <85 dBA.

Five studies (four cohort studies and one case-control study) with 149,911 participants from three WHO regions reported estimates of the effect of occupational exposure to noise on the risk of hypertension, compared with no occupational exposure to noise. Of these, three cohort studies were sufficiently homogenous to be included in a quantitative meta-analysis. As for the (over-) adjusted estimate they reported in a figure in their paper, it was below 1.00. Based on the pooling of the three remaining cohort studies, workers exposed to ≥85 dBA had 7% higher risk of acquiring hypertension (RR = 1.07, 95% CI 0.90 to 1.28, three studies, four estimates, 147,820 participants, I^2^ = 52%; Fig. 13). We found evidence of publication bias (major asymmetry in the Doi plot and LFK index = 4.07). Upon exclusion of each estimate one-at-a-time, the pooled RR remained non-significant. The fixed effects and IVhet models each yielded a slightly lower RR of 2% (n.s.)

The fourth cohort study, Huo Yung Kai et al. (2018), was not included in the meta-analysis, because we could only calculate an unadjusted estimate from raw data, and as per our protocol we did not combine adjusted and unadjusted effect estimates in the same meta-analysis. This study reported raw data from which we calculated an unadjusted RR of 1.27, with a 95% CI of 1.06–1.52. While this effect estimate was somewhat higher than that presented in the main analysis, we judged it to still be similar, considering that it was not adjusted for confounding, which may explain the differences found.

The case-control study (Tong et al., 2017), reporting an adjusted effect estimate of OR 1.64, 95% CI 1.23–2.20, was also left out of the meta-analysis. This effect estimate was again also somewhat higher than that presented in the main analysis; nevertheless, we judged it to be similar, considering that it was expressed as an OR.

### 4.5 Additional analyses

Further sensitivity analyses were not performed for data from the main meta-analysis with comparison between the group working exposed to ≥85 dBA, compared with <85 dBA.

### 4.6 Quality of evidence

#### 4.6.1 Acquired IHD (IHD incidence)

Regarding downgrading considerations, we did not have serious concerns regarding risk of bias in the body of evidence on this comparison for this outcome. We judged the risk of bias to be “probably low” in the exposure assessment domain across the whole body of evidence. The risk of bias was overwhelmingly “low” or “probably low” across other domains, especially in the prioritized evidence (Eriksson et al., 2018b, Ising et al., 1997, Kersten and Backe, 2015, Song, 2013, Virkkunen et al., 2005). Considering that a JEM for noise lacks precision on the individual worker-level but is often the best feasible approach for exposure assessment in large cohort studies and that it provides relevant exposure information on job-title level, we judged that that the overall risk of bias across the body of evidence for IHD incidence was “probably low”. Therefore, the overall quality of evidence was not downgraded (±0 levels). We had very serious concerns for indirectness of the evidence because the cut-off noise level defining the unexposed workers varied across studies and was not always exactly <85 dBA; for example, it was <75 dBA in Eriksson et al. (2018b) and <80 dBA in Virkkunen et al. (2005). Studies were limited to males only and one WHO region (Europe), and we could not rule out that the effect (if any) differs by one or both of sex and WHO region. Therefore, the quality of evidence was downgraded for very serious concerns for indirectness (−2 levels). We did not have any serious concerns regarding inconsistency (±0 levels). We also had no serious concerns for imprecision, given the narrow 95% CI of the pooled effect in the main meta-analysis (±0 levels). We could not formally assess publication bias with a funnel plot due to the small number of studies on this outcome, but effect estimates appeared to be relatively consistent across studies; therefore the quality of evidence was not downgraded as we had no serious concerns for this consideration (±0 levels).

Regarding upgrading domains, to judge downgrading for the consideration of a large effect size, we applied the WHO definition for a large effect on CVD for *environmental* exposure to noise: RR ≥ 1.25 (van Kempen et al., 2018). Since the pooled effect estimate from our main meta-analysis was an RR of 1.29 (95% CI 1.15–1.43), we upgraded the quality of evidence for this large effect estimate (+1 level). No upgrade was made for residual confounding (±0 levels). We investigated dose–response associations but did not find evidence for such a dose–response, given durations of exposure in only one study (±0 levels).

In conclusion, we started our assessment at “moderate quality of evidence” because the body of evidence comprised only observational studies. We downgraded by two levels (−2) for indirectness. We upgraded by one level (+1) for a large effect estimate. We arrived at a final rating of “low quality of evidence”: Further research is very likely to have an important impact on our confidence in the estimate of effect and is likely to change the estimate.

#### 4.6.2 Died from IHD (IHD mortality)

**Fig. 14:**
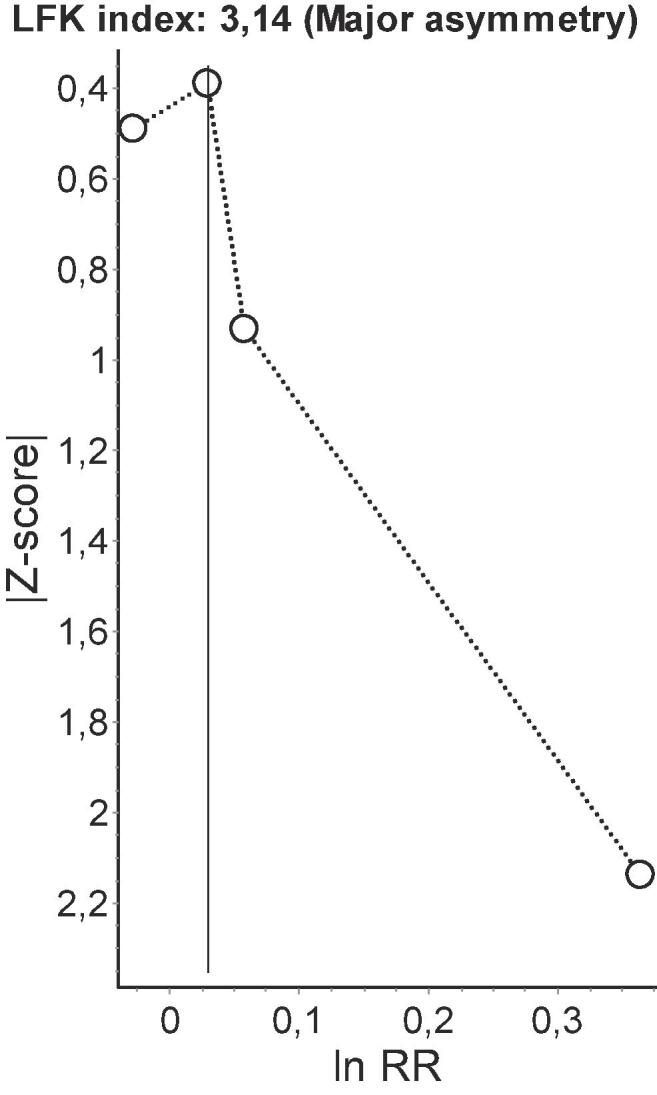
Doi plot of prioritized evidence (cohort studies), Outcome: Died from IHD (IHD mortality). Note. LFK – Luis Furuya-Kanamori index of asymmetry, RR – relative risk. Major Doi plot asymmetry indicated by the high LFK index is suggestive of possible publication bias.

Regarding downgrading considerations, we did not have serious concerns regarding risk of bias in the body of evidence on this comparison for this outcome (±0 levels). We judged the risk of bias to be “probably low” in the exposure assessment domain across the prioritized body of evidence. The large representative studies that contributed almost exclusively to the estimate of magnitude of effect (Davies, 2002, Pettersson et al., 2020) used a JEM, which, as argued above, provides informative exposure information despite its limitations on the individual level. We had very serious concerns for indirectness though, because the body of evidence had limitations in its population coverage (no females in three out of four studies in the main meta-analysis) and its exposure assessment (several studies used self-reported noise exposure and equating the exposure in Davies (2002) to our standard definition required certain assumptions). Therefore, we downgraded by two levels for this consideration (−2). We had serious concerns for neither inconsistency, nor imprecision (±0 levels). We had serious concerns for publication bias as our Doi plot suggested major asymmetry (Fig. 14) and therefore downgraded by one level (−1).

Regarding upgrading domains, we upgraded neither for a large effect estimate, nor residual confounding, nor evidence of a dose–response relationship (±0 levels).

In conclusion, we started at “moderate quality of evidence” due to all included studies being observational, we downgraded by a total of three levels (−3), did not upgrade (±0 levels), and consequently arrived at a final rating of “low quality of evidence”.

#### 4.6.3 Acquired stroke (stroke incidence)

Regarding downgrading considerations, we did not have serious concerns regarding risk of bias in the body of evidence on this comparison for this outcome (±0 levels). While one of the studies (Gopinath et al., 2011) carried a “high” risk of bias in the exposure assessment domain and a “probably high” rating in the outcome assessment, selective reporting and other bias domains, the other two studies (Eriksson et al., 2018b, Stokholm et al., 2013b), which contributed the bulk of prioritized evidence, were free of apparent bias. They were judged to be at “probably low” risk of bias in the exposure assessment domain because of using a JEM. Nevertheless, we had very serious concerns for indirectness, because the population covered by the body of evidence was limited to one WHO region and representative by neither sex, nor age, and also because the comparator was below the defined exposure limit; we therefore downgraded by two levels for indirectness (−2). We did not have any serious concerns regarding inconsistency in our main meta-analysis (±0 levels). We did have serious concerns however for imprecision, because the lower limit of the 95% CI of the pooled effect estimate indicated a small decrease in risk, whereas the upper limit indicated a large increase – major imprecision; we therefore downgraded by one level (−1). We could not test for publication bias with just three estimates, but from our qualitative assessment of these estimates, we did not have serious concerns for publication bias (±0 levels).

Regarding upgrading domains, we upgraded neither for a large effect estimate, nor a dose–response relationship, nor residual confounding (±0 levels).

In conclusion, we started at a rating of “moderate quality of evidence”, due to all included studies being observational, and downgraded by three levels (−3) and did not upgrade (±0 levels). Thus, our final rating was “low quality of evidence”.

#### 4.6.4 Died from stroke (stroke mortality)

Regarding downgrading considerations, we did not have serious concerns regarding risk of bias in the body of evidence on this comparison for this outcome (±0 levels). Of the three studies, two were effectively driving the observed effect (Davies, 2002, Pettersson et al., 2020) and they were deemed free of serious bias. They received a “probably low” rating in the exposure assessment domain due to using a JEM for noise. The other study (Gopinath et al., 2011) which carried “high” risk fo bias in the exposure assessment domain, had a negligible contribution toward the estimate of magnitude of effect. Still, we had very serious concerns for indirectness because the population excluded females in two of three studies, the exposure was subjective and self-reported in one study and capture of industrial sectors and occupations was either limited or unknown (−2 levels). We did not have any serious concerns regarding inconsistency as we judged the effect estimates across studies to be sufficiently homogeneous (±0 levels). We had serious concerns for imprecision given that the lower limit of the 95% CI from the pooled effect estimate indicated a small decrease in risk whereas the upper limit indicated a small to moderate increase (−1 level). We could not formally assess publication bias with a funnel plot since the body of evidence comprised three effect estimates only, but our qualitative assessment of these estimates raised no serious concerns, and we consequently did not downgrade for this consideration (±0 levels).

Regarding upgrading domains, we upgraded neither for a large effect estimate, nor a dose–response relationship, nor residual confounding (±0 levels).

In conclusion, we started at “moderate quality of evidence”, due to all included studies being observational, downgraded by a total of three levels (−3), and did not upgrade (±levels). Thus, we arrived at a final rating of “low quality of evidence”.

#### 4.6.5 Acquired hypertension (hypertension incidence)

**Fig. 15:**
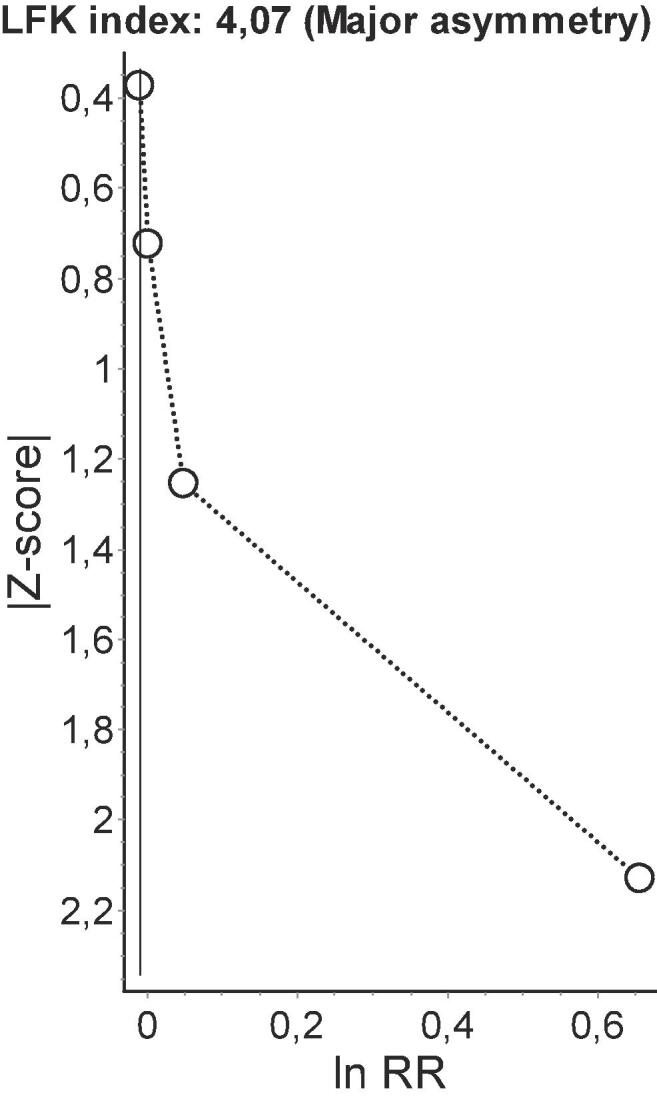
Doi plot of prioritized evidence (cohort studies), Outcome: Acquired hypertension (hypertension incidence). Note. LFK – Luis Furuya-Kanamori index of asymmetry, RR – relative risk. Major Doi plot asymmetry indicated by the high LFK index is suggestive of possible publication bias.

Regarding downgrading considerations, we did not have serious concerns regarding risk of bias in the body of evidence on this comparison for this outcome (±0 levels). The risk of bias was “probably high” in the selection bias (high attrition rate and systematic differences between included and dropout participants) and other bias domains (adjustment for mediators). However, the studies that supplied prioritized evidence (Chang et al., 2013, Stokholm et al., 2013a, Tessier-Sherman et al., 2017) were largely free of apparent bias that could seriously undermine our confidence in the observed effect of noise. Although, we had very serious concerns for indirectness because studies from the population excluded females in most studies, did not capture national populations, and only covered selected or unknown industrial sectors or occupations, as well as using different noise exposure cut-off levels to define the comparator (−2 levels). We did not have serious concerns for inconsistency (±0 levels). Imprecision raised serious concerns, as the lower limit of the 95% CI of the pooled effect estimate indicated a small decrease in risk, whereas the upper limit of the CI indicated a large increase; we therefore downgraded by one level (−1). We also had serious concerns for publication bias, because we interpreted the Doi plot (Fig. 15) as indicative of some asymmetry, while noting the limited assessment possible with only four studies included (−1 level).

Regarding upgrading domains, we did not upgrade for large effect size, nor for dose–response (±0 levels). However, we did upgrade by one level for residual confounding, because we considered the over-adjustment for mediators to have biased the effect estimates towards the null, so that residual confounding could potentially explain why we did not find evidence for an increased risk (+1 level).

In conclusion, we started at “moderate quality of evidence” for a body of evidence limited to observational studies, we downgraded by four levels (−4), we upgraded by one level (+1), and arrived at a final rating of “low quality of evidence”.

### 4.7 Assessment of strength of evidence

According to our protocol (Teixeira et al., 2019), we rated the strength of evidence based on a combination of four criteria outlined in the Navigation guide: (1) Quality of the entire body of evidence; (2) Direction of the effect estimate; (3) Confidence in the effect estimate; and (4) Other compelling attributes.

#### 4.7.1 Quality of the entire body of evidence

Concerning the number, size, and quality of individual studies, the body of evidence is sufficient to assess the toxicity/harmfulness of the exposure. The meta-analyses including a very large number of participants, and considering relevant confounders, documents a significantly increased risk (large effect) of incident IHD (acquiring IHD) when working exposed to ≥85 dBA compared with <85 dBA, with the lower limit of the 95% CI beyond 1.0 and a rather narrow overall 95% CI. For the other outcomes, the observed risk was slightly-to-moderately increased and non-significant, with the lower limit of the 95% CI below 1.0. We recognize the growing resistance by experts against formal categorization of findings into statistically significant or non-significant and we appreciate that the practical implications of all values inside a confidence interval should be of interest (Amrhein et al., 2019). The quality of most cohort studies (prioritized evidence) is adequate, given similar study protocols, consistent measurement of exposure and outcome, and clear temporal distinction between exposure and outcome. Overall, risk of bias of prioritized evidence is “probably low”, thus supporting adequate quality.

#### 4.7.2 Direction of the effect estimate

The study results are sufficient to assess the direction of the effect estimate. For all outcomes evaluated, no single study documented a significant negative effect estimate (with the higher CI below 1.0). Our incidence outcomes had great heterogeneity from 0% I^2^ for IHD and stroke and 52% for hypertension, while mortality-related outcomes had an I^2^ of 0–26%. The mortality studies accounted for acceptable consistency of findings.

#### 4.7.3 Confidence in the effect estimate

There is limited evidence to determine the level of confidence in the effect estimate, at least for the following reasons. First, while studies include the test of several relevant confounders that in part can also act as mediators, no additional data are reported in those studies on causal pathways linking exposure to the health outcome under study. Indirect supportive evidence comes from studies dealing with health-adverse working conditions other than occupational noise, but conditions that implicate identical pathways from exposure to outcome, such as adverse health behaviours or chronic psychosocial stress with pathophysiological effects on CVD. However, we take into account the compelling evidence that in the residential environment, even at levels much lower than 85 dBA, road traffic noise increases the risk of IHD (van Kempen et al., 2018). Second, the assumption of a dose–response relationship between noise levels and years of exposure and the outcome was difficult to determine from our findings. There was no indication of an effect at the lowest exposure category and perhaps a slightly larger effect at the next lowest exposure category. There could be a threshold, but this is difficult to ascertain from the currently available evidence. Third, the magnitude of the effect estimate was large only for IHD incidence, which raises our certainty in that effect, but the pooled RRs were < 1.25 for the other outcomes, according to the definition in the WHO evidence review on *environmental* exposure to noise and CVD (van Kempen et al., 2018). Still, we acknowledge that even a modest increase in (population-level) risk can be relevant for policy under conditions of high prevalence of the exposure (which is certainly the case with occupational exposure to noise). Fourth, no intervention studies are available that demonstrate a reduction of the effect estimate because of reducing the exposure to minimal level.

#### 4.7.4 Other compelling attributes

We were not able to access data that could offer evidence for a discussion of other compelling attributes in assessing the strength of evidence.

#### 4.7.5 Rating by outcome and comparison

Based on the considerations presented above, we judged the existing bodies of evidence as:

• Inadequate evidence for harmfulness for IHD prevalence and mortality; stroke prevalence, incidence and mortality; and hypertension prevalence, incidence and mortality.
• Limited evidence for harmfulness for IHD incidence; a positive relationship is observed between exposure and outcome where chance, bias, and confounding cannot be ruled out with reasonable confidence.

## 5 Discussion

### 5.1 Summary of evidence

As shown in the table of summary of findings (Table 5), our systematic review found low quality evidence of associations of occupational noise ≥85 dBA with elevated risk of acquiring IHD and concluded there was limited evidence of harmfulness from human evidence for acquiring IHD. For all other included outcomes, we found bodies of evidence that we rated as providing low quality of evidence and, in terms of strength of evidence, to be inadequate for us to determine harmfulness with any confidence. More research is needed to assess the effects of occupational exposure to noise on the prevalence, incidence and mortality from IHD, stroke and hypertension. Future research should use standardized, high-quality exposure and outcome assessments (definitions, measurements, etc) to ensure that more evidence that is comparable and harmonized becomes available for more comprehensive, quantitative meta-analysis.

**Table 5:** Summary of findings.

**Effect of occupational exposure to noise on cardiovascular disease among workers**					
**Population:** all ≥ 15 years workers **Settings:** all countries and work settings **Exposure:** occupational exposure to noise (defined as ≥85 dBA) **Comparator:** no occupational exposure to noise (defined as <85 dBA)					

**Outcomes**	**Relative effect (95% CI)**	**No. of participant (studies)**	**Navigation Guide quality of evidence rating**	**Navigation Guide strength of evidence rating for human evidence**	**Comments**

**IHD prevalence**				Inadequate evidence for harmfulness	No eligible studies found.
**IHD incidence**	**–**x	11,758 (2 studies)	⊕⊝⊝ Low^a^^,^^b^	Limited evidence of harmfulness	Better indicated by lower values. The available evidence is sufficient to determine the effects of the exposure, but confidence in the estimate is constrained. As more information becomes available, the observed effect could change, and this change may be large enough to alter the conclusion. A positive relationship is observed between exposure and outcome where chance, bias, and confounding cannot be ruled out with reasonable confidence.
**IHD mortality**	**–**x	198,926 (4 studies)	⊕⊝⊝ Low^a^^,^^c^	Inadequate evidence of harmfulness	Better indicated by lower values. Studies permit no conclusion about a toxic effect. The available evidence is insufficient to assess effects of the exposure. More information may allow an estimation of effects.
**Stroke prevalence**				Inadequate evidence for harmfulness	No eligible studies found.
**Stroke incidence**	**–**x	170,000 (2 studies)	⊕⊝⊝ Low^a^^,^^d^	Inadequate evidence of harmfulness	Better indicated by lower values. Studies permit no conclusion about a toxic effect. The available evidence is insufficient to assess effects of the exposure. More information may allow an estimation of effects.
**Stroke mortality**	**–**x	195,539 (3 studies)	⊕⊝⊝ Low^a^^,^^d^	Inadequate evidence of harmfulness	Better indicated by lower values. Studies permit no conclusion about a toxic effect. The available evidence is insufficient to assess effects of the exposure. More information may allow an estimation of effects.
**Hypertension prevalence**				Inadequate evidence for harmfulness	No eligible studies found.
**Hypertension incidence**	**–**x	147,820 (3 studies/4 estimates)	⊕⊝⊝ Low^a^^,^^d^^,^^c^^,^^e^	Inadequate evidence of harmfulness	Better indicated by lower values. Studies permit no conclusion about a toxic effect. The available evidence is insufficient to assess effects of the exposure. More information may allow an estimation of effects.
**Hypertension mortality**				Inadequate evidence for harmfulness	No eligible studies found.

**CI**: confidence interval; **RR**: relative risk.

*Navigation Guide quality of evidence ratings:*

**High quality:** Further research is very unlikely to change our confidence in the estimate of effect.

**Moderate quality:** Further research is likely to have an important impact on our confidence in the estimate of effect and may change the estimate.

**Low quality:** Further research is very likely to have an important impact on our confidence in the estimate of effect and is likely to change the estimate.

*Navigation Guide strength of evidence ratings:*

**Sufficient evidence of toxicity/harmfulness**: The available evidence usually includes consistent results from well‐designed, well‐conducted studies, and the conclusion is unlikely to be strongly affected by the results of future studies. For human evidence a positive relationship is observed between exposure and outcome where chance, bias, and confounding, can be ruled out with reasonable confidence.

**Limited evidence of toxicity/harmfulness**: The available evidence is sufficient to determine the effects of the exposure, but confidence in the estimate is constrained by such factors as: the number, size, or quality of individual studies, the confidence in the effect, or inconsistency of findings across individual studies. As more information becomes available, the observed effect could change, and this change may be large enough to alter the conclusion. For human evidence a positive relationship is observed between exposure and outcome where chance, bias, and confounding cannot be ruled out with reasonable confidence.

**Inadequate evidence of toxicity/harmfulness**: Studies permit no conclusion about a toxic effect. The available evidence is insufficient to assess effects of the exposure. Evidence is insufficient because of: the limited number or size of studies, low quality of individual studies, or inconsistency of findings across individual studies. More information may allow an estimation of effects.

**Evidence of lack of toxicity/harmfulness**: The available evidence includes consistent results from well‐designed, well‐conducted studies, and the conclusion is unlikely to be strongly affected by the results of future studies. For human evidence more than one study showed no effect on the outcome of interest at the full range of exposure levels that humans are known to encounter, where bias and confounding can be ruled out with reasonable confidence. The conclusion is limited to the age at exposure and/or other conditions and levels of exposure studied.

x Because we are very uncertain about the effect estimate, we do not present it in this summary of findings table.

a Downgraded by two level (−2) for very serious concerns for indirectness.

b Upgraded by one level (+1) for large effect size (defined as RR ≥ 1.25).

c Downgrade by one level (−1) for serious concerns for publication bias.

d Downgrade by one level (−1) for serious concerns for imprecision.

e Upgrade by one level (+1) for residual confounding.

### 5.2 Comparison to previous systematic review evidence

Five previous systematic reviews and meta-analyses (Domingo-Pueyo et al., 2016, Dzhambov and Dimitrova, 2016, Hwang and Hong, 2012, Skogstad et al., 2016, van Kempen et al., 2002) and one after protocol published (Yang et al., 2018) have lent support to the notion that occupational exposure to noise is associated with a modestly increased risk of morbidity or mortality from one or more CVDs. Our systematic review and meta-analysis partially corroborates previous systematic reviews and meta-analytic evidence, but only for one outcome, IHD incidence, and not for any of the other eight CVD outcomes included in this systematic review.

First, previous systematic reviews and meta-analyses did not similarly define the exposure and/or outcome but rather considered any occupational exposure to noise without dose differentiation and any CVD or group of CVDs (e.g., both IHD and stroke). Some previous systematic reviews included cross-sectional, cohort and case-control studies and combined cross-sectional studies with analytic studies (cohort and case-control), whereas we excluded cross-sectional studies and only included analytic studies that can provide information on causal relationships. Our meta-analysis included a consistent definition of categories of occupational noise and identified studies from different WHO regions.

Second, the Skogstad et al. (2016) meta-analysis included 12 prospective cohort studies from high-income countries published between 1999 and 2013, most of which were judged to be of high quality, but with some methodological shortcomings in exposure assessment. This study represents the most comprehensive systematic review of analytic studies on the topic up to the year of its publication, and its major strength is the inclusion of published and unpublished studies (thus addressing publication bias). However, the analytic approach and data extraction have been scrutinized (Dzhambov and Dimitrova, 2016).

Being compared with these recent comprehensive systematic reviews, our systematic review and meta-analysis has the following additional strengths. First, we expanded the search, in terms of both timeframe and language of the retrieved publications. Second, we extended the types of eligible study designs by considering non-randomized intervention studies. None of the previous systematic reviews and meta-analyses distinguished nine outcomes s as we did, namely prevalence, incidence and mortality for each of hypertensive heart disorder, IHD and stroke, respectively; our systematic review thereby adds accuracy. Finally, we adopted a set of modern analytical techniques to check the robustness of our findings. In summary, our systematic review builds on the important work of the previous systematic reviews and further updates, extends and differentiates the existing body of systematic review evidence.

### 5.3 Limitations and strengths of this systematic review

#### 5.3.1 Limitations

Our systematic review has several limitations. First, the number of effects estimates per meta-analysis was low; therefore, we could not conduct subgroup analyses, nor meta-regression. No disaggregation by country, sex, age group, industrial sector, and occupation was possible. In some cases, that also prevented us from assessing publication bias.

Second, some studies (Gopinath et al., 2011, Huo Yung Kai et al., 2018, Ising et al., 1997, Suadicani et al., 2012) used self-reported measures of occupational noise exposure, which may be prone to recall bias or be reciprocally related to CVD. Nevertheless, standardized questions on the vocal effort needed to overcome ambient noise are considered valid proxies for a noise level >85 dBA (Ahmed et al., 2004, Neitzel et al., 2016, Neitzel et al., 2011, Schlaefer et al., 2009).

Third, although the exposed groups across studies were largely comparable, in some studies (Chang et al., 2013, Eriksson et al., 2018b, Kersten and Backe, 2015, Stokholm et al., 2013a, Stokholm et al., 2013b) the cut-off noise level was below 85 dBA. A lower reference group (e.g., <75 dBA) could have resulted in an inflation of the respective relative risk of an unknown size. Still, these studies were retained as nominally they fulfilled the predefined inclusion criterion in the systematic review protocol, namely that the control group should be exposed to <85 dBA.

Fourth, from some studies comparing two (or more) noise-exposed groups (≥85 dBA) with the same unexposed (control) group (Stokholm et al., 2013a), we had to extract only one estimate because they did not report all needed raw data to compute a composite study-level effect size. From other studies with two (or more) noise-exposed groups, some of which below the 85 dBA cut off (Chang et al., 2013, Virkkunen et al., 2005), we used only the estimate for the group exposed to ≥85 dBA. That resulted in information loss from the other group. Another related potential limitation is that in some studies (e.g., Davies (2002)) the exposed – unexposed contrast was defined by differences in duration of noise exposure rather than differences in noise intensity (e.g., ≥85 dBA for >3 years vs. >85 dBA for <3 years). Since the authors of those studies could not re-analyse their data as requested, we decided to include those studies making an expert judgement that very short duration of exposure would effectively equate to no exposure (in terms of CVD risk). Still, that could have attenuated the observed risk. A detailed list of further potential sources of bias in specific effect estimates and justification of our decision to include them can be found in Appendix 3 in the Supplementary data.

Fifth, sufficiently homogeneous studies meeting inclusion criteria were pooled together irrespective of the risk of bias associated with them. This approach was adopted with a view to recent concerns that stratification by study quality may introduce a form of selection bias in meta-analyses (Stone et al., 2019)

Finally, we did not receive some missing data we requested for the studies included in this systematic review. We requested missing data from principal study authors at least three times, but the principal study authors did not share these requested missing data with us.

#### 5.3.2 Strengths

Our systematic review and meta-analysis have a number of strengths, including:

• Previous systematic reviews have not clarified whether all the steps of a systematic review have been performed, but our systematic review and meta-analysis have done so, including we being pre-published a protocol and assessed strength of evidence of the protocol, which represents a substantial improvement in the systematic review of methods on the subject.
• Previous systematic reviews have not sought to differentiate IHD incidence and IHD mortality, stroke incidence and mortality and hypertension, but our systematic review improves accuracy by differentiating these different outcomes.
• Previous systematic reviews have not comprehensively provided detailed account of all analytic steps of the systematic review and meta-analysis for comparisons of standard categories of occupational exposure to noise ≥85 dBA, compared with <85 dBA, and again this provides an improvement in accuracy of systematic review evidence on this topic.
• Whereas previous systematic review evidence has not comprehensively assessed risk of bias and quality of evidence using established systematic review frameworks with dedicated tools and approaches, we have rigorously applied the Navigation Guide framework in this systematic review, which should have ensured rigor and transparency in this systematic review.
• In previous systematic reviews, strength of the evidence was not commonly assessed, but in our systematic review, we have applied pre-specified criteria to rate the strength of evidence for each included comparison for each included outcome, and this is another novel contribution to the synthetic body of evidence on the topic.
• Finally, to our knowledge, this is the first systematic review and meta-analysis conducted specifically for a global occupational burden of disease study, and as such it provides a model for future systematic reviews that will help ensure that these global health estimates adhere fully with the *GATHER Guidelines for Accurate and Transparent Health Estimates Reporting* (Stevens et al., 2016).

## 6 Use of evidence for burden of disease estimation

This systematic review and meta-analysis was conducted by WHO and ILO, supported by a large number of experts, for the development of the WHO/ILO Joint Estimates (Ryder, 2017). More specifically, it aimed to provide the crucial evidence base for the organizations to consider producing estimates of the burden of deaths and DALYs from CVDs (i.e. IHD and stroke) attributable to occupational exposure to noise.

This systematic review found limited evidence for harmfulness of occupational exposure to noise (≥85 dBA) for IHD incidence and inadequate evidence for harmfulness for the other included outcomes: IHD prevalence, IHD mortality, stroke prevalence, stroke incidence, stroke mortality, hypertension prevalence, hypertension incidence, and hypertension mortality (Table 5). Producing estimates of the burden of CVDs attributable to occupational exposure to noise (≥85 dBA) appears neither evidence-based nor warranted, and the parameters reviewed (including the pooled RRs from the meta-analyses for these comparisons) appear not suitable as input data for WHO/ILO modelling of work-related burden of disease and injury.

## 7 Conclusions

For acquiring IHD, we judged the existing body of evidence from human data to provide “limited evidence of harmfulness”; a positive relationship is observed between exposure and outcome where chance, bias, and confounding cannot be ruled out with reasonable confidence. For all other outcomes, the bodies of evidence were judged as “inadequate evidence of harmfulness”. Producing estimates for the burden of CVD attributable to occupational exposure to noise appears to not be evidence-based at this time.

## 8 Differences between protocol and systematic review

• We were unable to search the International Clinical Trials Register Platform, Toxline and Health and Environmental Research Online (HERO).
• The original search strategy was reviewed and modified to make it clear, sensitive and more efficient. New keywords and subject headings were added, some descriptors were exploded. We also use more wildcards and we expanded the strategy to identify the appropriate study designs.

## Financial support

All authors are salaried staff members of their respective institutions. The publication was prepared with financial support from the World Health Organization cooperative agreement with the Centres for Disease Control and Prevention National Institute for Occupational Safety and Health of the United States of America (Grant 1E11OH0010676-02; Grant 6NE11OH010461-02-01; and Grant 5NE11OH010461-03-00); the German Federal Ministry of Health (BMG Germany) under the BMG-WHO Collaboration Programme 2020–2023 (WHO specified award ref. 70672); and the Spanish Agency for International Cooperation (AECID) (WHO specified award ref. 71208).

## Sponsors

The sponsors of this systematic review are the World Health Organization and the International Labour Organization.

## Author contributions

Had the idea for this systematic review: FP, Ivan Ivanov (WHO), Nancy Leppink (ILO)
Selected the lead reviewers and gathered the review teams: FP, Ivan Ivanov, Nancy Leppink
Coordinated the entire series of systematic reviews: FP, Yuka Ujita (ILO)
Were the lead reviewers of this systematic review: LT, DG
Led the design of the systematic review including developed the standard methods: FP
Contributed substantially to the design of the systematic review: LT, AB, DTCS, EG, MPL, JS, DG
Conducted the search: LT, AB, DTCS, EG, MSMS, MPL, JS, DG
Selected studies: LT, AMD, AB, DTCS, EG, KH, SI, MPL, BMR, JS, AV, DG
Extracted data: LT, AMD, AB, EG, KH, SI, MPL, BMR, JS, AV, DG
Requested missing data: LT, AMD, DTCS,
Assessed risk of bias: AMD, SI, BMR, AV, DG
Conducted the meta-analyses: LT, FP, AMD, BMR, DG
Assessed quality of evidence: LT, FP, AMD, DG
Assessed strength of evidence: LT, FP, AMD, DG
Developed the standards and wrote the template for all systematic reviews in the series: FP
Wrote the first draft of the manuscript using the template: LT, AMD, DG
Revised the manuscript critically for important intellectual content: All authors
Ensured tailoring of the systematic review for WHO/ILO estimation purposes: FP
Ensured harmonization across systematic reviews in the series: FP
Approved the final version of the systematic review to be published: All authors
Agreed to be accountable for all aspects of the work in ensuring that questions related to the accuracy or integrity of any part of the work are appropriately investigated and resolved: All authors.

## Declaration of Competing Interest

The authors declare that they have no known competing financial interests or personal relationships that could have appeared to influence the work reported in this paper.
