# Supplementary material for: The effect of occupational exposure to noise on ischaemic heart disease, stroke and hypertension: A systematic review and meta-analysis from the WHO/ILO Joint Estimates of the Work-Related Burden of Disease and Injury
Source: Environ Int. 2021 Sep;154:106387. doi: 10.1016/j.envint.2021.106387 (PMC8204276; doi:10.1016/j.envint.2021.106387)
Supplement: Supplementary data 1 [file mmc1.docx]

***Appendix 1 Search strategies for electronic academic databases***

| DATABASE/Date | STRATEGIES |
| --- | --- |
| Pubmed  March 21^st^, 2019. Updated on January 31^st^, 2020 | 1. Noise, Occupational[MESH] OR ((noise*[TIAB] OR noisy[TIAB] OR sound[TIAB] OR loud[TIAB]) AND (occupation*[TIAB] OR work*[TIAB] OR employ*[TIAB] OR labour*[TIAB] OR labor*[TIAB] OR job[TIAB])) 2. Cardiovascular Diseases[MESH] OR (("heart"[TIAB] OR "cardiac"[TIAB] OR "coronary"[TIAB]) AND "disease"[TIAB]) OR (CHD*[TIAB] NOT (Heart Defects, Congenital[MESH] OR “Congenital Heart disease”[TIAB])) OR Cardiovascular[TIAB] OR angina*[TIAB] OR cvd[TIAB] OR myocardial[TIAB] OR ischemi*[TIAB] OR ischaemi*[TIAB] OR Ischemia[MESH] OR infarct*[TIAB] OR stroke* OR stroke[MESH] OR cardiomyopath*[TIAB] OR myocardit*[TIAB] OR endocardit*[TIAB] OR Thrombosis[MESH] OR "Cardiovascular risk factors” [TIAB] OR (arrhythmi*[TIAB] AND cardiac[TIAB]) OR Hypertension[MeSH] OR Hypertens*[TIAB] OR (“Blood Pressure”[TIAB] AND High[TIAB]) 3. "Epidemiologic Studies"[Mesh] OR "Clinical Trial" [Publication Type] OR "Observational Study" [Publication Type] OR "Comparative Study" [Publication Type] OR Trial[TW] OR random*[TW] OR experiment*[TW] OR ((intervention[TW] OR observational[TW] OR epidemiologic*[TW] OR panel*[TW] OR “follow up”[TIAB] OR exposure*[TW]) AND (study[TIAB] OR studies[TIAB] OR analys*[TIAB])) OR Cohort*[TW] OR longitudinal*[TW] OR retrospective*[TW] OR prospective*[TW] OR "time series"[TIAB] OR before-after[TIAB] 4. Seroepidemiologic Studies[MESH] OR Cross-Sectional Studies[MESH] OR “cross sectional”[TIAB] 5. #1 and #2 and (#3 not #4) |
| Medline/Ovid  March 21^st^, 2019. Updated on January 31^st^, 2020 | 1. Noise, Occupational/ 2. ((noise* or noisy or loud or sound*) and (occupation* or work* or employ* or labour* or labor* or job)).mp. 3. 1 or 2 4. exp Cardiovascular Diseases/ 5. ((heart or cardiac or coronary) adj4 disease*).mp. 6. chd*.tw. not (Heart Defects, Congenital/ or 'Congenital Heart disease'.mp.) 7. (Cardiovascular or angina* or cvd or myocardial or isch*emi* or infarct*).mp. 8. (arrhythmi* adj3 cardiac).mp. 9. Stroke/ or stroke*.mp. 10. (cardiomyopath* or myocardit* or endocardit* or 'Cardiovascular risk factors' or hypertens* or 'high blood pressure').mp. 11. exp Ischemia/ or exp Thrombosis/ or exp Hypertension/ 12. 4 or 5 or 6 or 7 or 8 or 9 or 10 or 11 13. exp Epidemiologic Studies/ or Clinical Trial/ or Observational Study/ or Comparative Study/ 14. (Trial or experiment*).tw. 15. ((intervention or observational or epidemiologic* or panel* or 'follow up' or exposure) adj (study or studies or analys* or data)).tw. 16. (longitudinal* or retrospective* or prospective*).tw. 17. (random* or 'time series' or before-after).mp. [mp=title, abstract, original title, name of substance word, subject heading word, floating sub-heading word, keyword heading word, organism supplementary concept word, protocol supplementary concept word, rare disease supplementary concept word, unique identifier, synonyms] 18. 13 or 14 or 15 or 16 or 17 19. exp Seroepidemiologic Studies/ or Cross-Sectional Studies/ or 'cross sectional'.tw. 20. 18 not 19 21. 3 and 12 and 20 |
| Embase  March 29^th^, 2019 | - 1. ('occupational health'/exp AND 'noise'/exp) OR 'industrial noise'/exp/mj or 'noise pollution'/exp)   2. 'noise*':ti,ab,kw OR 'noisy':ti,ab,kw OR 'loud':ti,ab,kw   3. 'occupation*':ti,ab,kw OR 'work*':ti,ab,kw OR 'employ*':ti,ab,kw OR 'labour*':ti,ab,kw OR 'labor*':ti,ab,kw OR 'job':ti,ab,kw   4. 2 AND 3   5. 1 OR 4   6. 'cardiovascular disease'/exp/mj   7. ('(heart':ti,ab,kw OR 'cardiac':ti,ab,kw OR 'coronary)':ti,ab,kw) AND 'disease':ti,ab,kw   8. 'cardiovascular':ti,ab,kw OR 'angina*':ti,ab,kw OR 'cvd':ti,ab,kw OR 'myocardial':ti,ab,kw OR 'ischemi*':ti,ab,kw OR 'ischaemi*':ti,ab,kw OR 'ischemia':ti,ab,kw OR 'infarct*':ti,ab,kw OR 'stroke*':ti,ab,kw OR 'stroke':ti,ab,kw OR 'cardiomyopath*':ti,ab,kw OR 'myocardit*':ti,ab,kw OR 'endocardit*':ti,ab,kw OR 'thrombosis':ti,ab,kw   9. 'hypertens*':ti,ab,kw OR '(´blood pressure´and (high OR elevated)':ti,ab,kw   10. 'cardiac arrythmia':ti,ab,kw   11. 6 OR 7 OR 8 OR 9 OR 10   12. 5 AND 11   13. (filter Study types) ('clinical article'/de OR 'clinical trial'/de OR 'cohort analysis'/de OR 'comparative study'/de OR 'controlled study'/de OR 'human experiment'/de OR 'major clinical study'/de OR 'prospective study'/de OR 'randomized controlled trial'/de OR 'retrospective study'/de)   14. 12 AND 13   15. 14 AND [embase]/lim NOT ([embase]/lim AND [medline]/lim) |
| Web of Science  March 29^nd^, 2019 | 1. TS=(“Occupational health” and “noise”) OR TS=(“industrial noise” or “noise pollution") 2. TS=(noise* OR noisy OR loud) 3. TS=(occupation* OR work* OR employ* OR labour* OR labor* OR job) 4. 2 and 3 5. 1 or 4 6. TS=(cardiovascular disease OR ((heart OR cardiac OR coronary) AND disease)) 7. TS=(cardiovascular OR angina* OR cvd OR myocardial OR ischemi* OR ischaemi* OR ischemia OR infarct* OR stroke* OR stroke OR cardiomyopath* OR myocardit* OR endocardit* OR thrombosis OR hypertens*OR (blood pressure and (high or elevated)) OR "cardiac arrythmia") 8. 6 or 7 9. 5 and 8 10. TS=(“clinical article” OR “clinical trial” OR cohort OR “comparative study” OR “controlled study” OR “human experiment” OR “clinical study” OR prospective OR “randomized controlled trial” OR retrospective OR random* OR case-control OR non-randomized OR quasi-randomized OR before-after OR “BEFORE AND AFTER” OR “time series”) 11. 9 AND 10 |
| Scopus  April 1^st^, 2019 | 1. (TITLE-ABS-KEY (("Occupational health" AND "noise")) OR TITLE-ABS-KEY (("industrial noise" OR "noise pollution"))) = 22.211 2. TITLE-ABS-KEY (noise* OR noisy) W/8 TITLE-ABS-KEY (occupation* OR work* OR employ* OR labour* OR labor* OR job) = 37,441 3. 1 or 2 = 54.209 4. (TITLE-ABS-KEY ("cardiovascular diseases" ) ) OR ( TITLE-ABS-KEY ( heart OR cardiac OR coronary ) W/6 disease* ) = 899,853 5. (TITLE-ABS-KEY ( "blood pressure" ) W/6 TITLE-ABS-KEY ( high OR elevated ) ) OR ( TITLE-ABS-KEY ( cardiovascular OR angina* OR cvd OR myocardial OR ischemi* OR ischaemi* OR ischemia OR infarct* OR stroke* OR stroke OR cardiomyopath* OR myocardit* OR endocardit* OR thrombosis OR hypertens* OR "cardiac arrythmia" ) ) = 2,790.688 6. 4 or 5 = 2,998,699 7. 3 and 6 = 1,420 8. (TITLE-ABS-KEY ( "clinical article" OR "clinical trial" OR cohort OR "comparative study" OR "controlled study" OR "human experiment" OR "clinical study" OR prospective OR "randomized controlled trial" OR retrospective ) OR TITLE-ABS-KEY ( random* OR case-control OR non-randomized OR quasi-randomized OR before-after OR "BEFORE AND AFTER" OR "time series" ) ) 9. 7 and 8 = 561 |
| Lilacs  April 1^st^, 2019 | ((mh:("Ruido Ocupacional")) OR (mh:("Saúde do Trabalhador")) AND (tw:(ruido OR barulho OR barullo OR noise OR noisy OR sound OR sonido OR som)) OR ((tw:("Occupational health" OR “saúde ocupacional” OR “saúde do trabalhador” OR “salud ocupacional” OR “salud del trabajador” OR “salud laboral”)) AND (tw:(ruido OR barulho OR barullo OR noise OR noisy OR sound OR sonido OR som))) OR ((tw:(ruido OR barulho OR barullo OR noise OR noisy OR sound OR sonido OR som)) AND (tw:(occupation* OR work* OR employ* OR labour* OR labor* OR job)))) AND ((tw:(heart OR cardiologica OR cardiac* OR coronary OR coronaria* OR cardiovascular*) AND tw:(disease* OR doença* OR enfermedad*)) OR (tw:(hipertensão OR hipertension OR hypertension OR cardiovascular* OR angina* OR cvd OR dcv OR myocardial OR miocardiaca OR ischemi* OR ischaemi* OR isquemi* OR infarto OR infarct* OR “avc” OR “acidente vascular cerebral” OR “accidente cerebrovascular” OR stroke OR cardiomyopath* OR cardiomiopatia OR miocardit* OR myocardit* OR endocardit* OR thrombosis OR trombos* OR “arritmia cardiaca” OR "cardiac arrythmia"))) |

**Appendix 2 Description of missing data requested and received**

| **Study ID** | **Description of missing data** | **Person(s) from whom missing data were requested** | **Date of request(s)** | **Data received** |
| --- | --- | --- | --- | --- |
| Virkkunen et al. (2006) | Other cut-off: low-exposure (<85 dBA) | Hanna Virkkunen, University of Tampere, Finland | August 30, 2019 | September 20, 2019 (Not accessible through email and personal contacts) |
| Pettersson 2020 | Other cut-off - low-exposure (≤ 85 dBA); estimates adjusted only for relevant confounders | Hans Pettersson, Umeå University, Sweden | January 23, 2020 | January 24, 2020 |

***Appendix 3 Technical calculation notes***

*Table A3.1 Combining across multiple comparisons within a single study*

| **Study ID** | **Comparisons** | **Sample size** | **RR** | **LCI** | **UCI** | **lnRR** | **Variance of lnRR in group** | **Weighted lnRR in group** | **Weighted average lnRR for the study** | **Matrix of pairwise group comparisons** | **Matrix of pairwise correlations between the estimates** | **Variance of mean lnRR for the study** | **Average expRR for the study** | **expLCI** | **expUCI** |
| --- | --- | --- | --- | --- | --- | --- | --- | --- | --- | --- | --- | --- | --- | --- | --- |
| Davies (2002) – IHD | Control/reference group (< 3 years) | 123 | 1 |  |  |  |  |  | -0.028 | 3-10 and 10-20 years | 0.366 | 1.802 | 0.973 | 0.887 | 1.066 |
|  | 3-10 vs. < 3 years | 209 | 1.03 | 0.82 | 1.28 | 0.030 | 4.284 | 0.012 |  | 3-10 and 20-30 years | 0.397 |  |  |  |  |
|  | 10-20 vs. < 3 years | 217 | 0.98 | 0.76 | 1.22 | -0.020 | 4.956 | -0.008 |  | 3-10 and > 30 years | 0.442 |  |  |  |  |
|  | 20-30 vs. < 3 years | 165 | 0.86 | 0.68 | 1.09 | -0.151 | 4.173 | -0.053 |  | 10-20 and 20-30 years | 0.392 |  |  |  |  |
|  | > 30 vs. < 3 years | 102 | 0.8 | 0.61 | 1.05 | -0.223 | 4.319 | -0.062 |  | 10-20 and > 30 years | 0.435 |  |  |  |  |
|  |  |  |  |  |  |  |  |  |  | 20-30 and >30 years | 0.480 |  |  |  |  |
|  |  |  |  |  |  |  |  |  |  |  |  |  |  |  |  |
| Davies (2002) – Stroke | Control/reference group (< 3 years) | 48 | 1 |  |  |  |  |  | 0.006 | 3-10 and 10-20 years | 0.347 | 1.722 | 1.006 | 0.880 | 1.149 |
|  | 3-10 vs. < 3 years | 90 | 1.15 | 0.81 | 1.63 | 0.140 | 4.392 | 0.052 |  | 3-10 and 20-30 years | 0.347 |  |  |  |  |
|  | 10-20 vs. < 3 years | 91 | 1.05 | 0.74 | 1.51 | 0.049 | 4.601 | 0.018 |  | 3-10 and > 30 years | 0.402 |  |  |  |  |
|  | 20-30 vs. < 3 years | 91 | 1.01 | 0.71 | 1.44 | 0.010 | 4.523 | 0.004 |  | 10-20 and 20-30 years | 0.345 |  |  |  |  |
|  | > 30 vs. < 3 years | 53 | 0.83 | 0.56 | 1.24 | -0.186 | 4.153 | -0.050 |  | 10-20 and > 30 | 0.4 |  |  |  |  |
|  |  |  |  |  |  |  |  |  |  | 20-30 and >30 years | 0.4 |  |  |  |  |
|  |  |  |  |  |  |  |  |  |  |  |  |  |  |  |  |
| Suadicani (2012) | Control/reference group (0 years) | 1890 | 1 |  |  |  |  |  | -0.178 | 1-4 and ≥5 years | 0.773 | 217.323 | 0.837 | 0.494 | 1.418 |
|  | 1-4 vs. 0 years | 100 | 0.56 | 0.21 | 1.53 | -0.580 | 510.743 | -0.385 |  |  |  |  |  |  |  |
|  | ≥ 5 vs. 0 years | 1008 | 1.03 | 0.76 | 1.39 | 0.030 | 68.742 | 0.029 |  |  |  |  |  |  |  |
|  |  |  |  |  |  |  |  |  |  |  |  |  |  |  |  |
| Kersten (2015) – Men | Control/reference group (46-61 dBA) | 818 | 1 |  |  |  |  |  | 0.054 | 85-94 and 95-124 dBA | 0.822 | 45.59 | 1.055 | 0.717 | 1.1.554 |
|  | 85-94 dBA | 300 | 0.61 | 0.46 | 0.8 | -0.49 | 22.28 | -0.47 |  |  |  |  |  |  |  |
|  | 95-124 dBA | 54 | 2.18 | 1.17 | 4.05 | 0.779 | 87.50 | 0.58 |  |  |  |  |  |  |  |
|  |  |  |  |  |  |  |  |  |  |  |  |  |  |  |  |
| Ising (1997) | Control/reference group (refrigerator/ typewriter) | 1370 | 1 |  |  |  |  |  | 0.531 | Lawn-mower and electric drill | 0.750 | 29.895 | 1.701 | 1.375 | 2.104 |
|  | Electric lawn-mower | 468 | 1.4 | 1.03 | 1.97 | 0.336 | 50.30 | 0.243 |  | Lawn-mower and pneumatic drill | 0.790 |  |  |  |  |
|  | Electric drill | 446 | 2 | 1.45 | 2.74 | 0.693 | 47.86 | 0.495 |  | Electric drill and pneumatic drill | 0.795 |  |  |  |  |
|  | Pneumatic drill | 259 | 3.8 | 2.68 | 5.44 | 1.335 | 53.13 | 0.855 |  |  |  |  |  |  |  |
|  |  |  |  |  |  |  |  |  |  |  |  |  |  |  |  |
| Tessier Sherman (2017) | Control/reference group (< 82 dBA) | 950 | 1 |  |  |  |  |  | 0.001 | 82-84.99 and 85-87.99 dBA | 0.688 | 39.877 | 1.001 | 0.761 | 1.315 |
|  | 82-84.99 dBA | 522 | 1.01 | 0.7 | 1.45 | 0.010 | 50.802 | 0.007 |  | 82-87.99 and >88 dBA | 0.714 |  |  |  |  |
|  | 85-87.99 dBA | 341 | 1.01 | 0.63 | 1.61 | 0.010 | 73.962 | 0.006 |  | 85-87.99 and ≥ 88 dBA | 0.766 |  |  |  |  |
|  | ≥ 88 dBA | 239 | 0.98 | 0.58 | 1.65 | -0.020 | 84.578 | -0.012 |  |  |  |  |  |  |  |

Abbreviations: RR – relative risk, LCI/UCI – lower/upper 95% confidence interval limit, ln – natural logarithm, exp – exponential estimate.

*Table A3.2 Input data for main meta-analysis of prioritized evidence (cohort studies), Outcome: Acquired ischaemic heart disease (IHD incidence)*

| **Study ID** | **Risk estimate** | **Effect size** | **LCI** | **UCI** | **Pooled effect size if study excluded** | **Comparison reported in study** | **Notes** |
| --- | --- | --- | --- | --- | --- | --- | --- |
| Eriksson (2018) | HR | 1.27 | 0.99 | 1.63 | 1.29 (1.15. 1.46) | ≥ 85 vs. < 75 dBA | Age-adjusted |
| Virkkunen (2005) | HR | 1.29 | 1.15 | 1.46 | 1.27 (0.99. 1.63) | ≥ 80/85 vs. < 80 dBA | The estimate from longest follow-up and continuous exposure was used (the other follow-ups yield similar effect estimates) |

Abbreviations: HR – hazard ratio. LCI/UCI – lower/upper 95% confidence interval limit.

*Table A3.3 Input data for main meta-analysis of prioritized evidence (cohort studies), Died from ischaemic heart disease (IHD mortality)*

| **Study ID** | **Risk estimate** | **Effect size** | **LCI** | **UCI** | **Pooled effect size if study excluded** | **Comparison reported in study** | **Notes** |
| --- | --- | --- | --- | --- | --- | --- | --- |
| Davies (2002) | Composite RR | 0.973 | 0.887 | 1.066 | 1.09 (0.96. 1.23) | ≥85dBA for > 3 years vs. > 85dBA for < 3 years |  |
| Gopinath (2011) | HR | 1.44 | 0.94 | 2.12 | 1.00 (0.93. 1.08) | Unable to hear speech vs. None |  |
| Pettersson (2020) | RR | 1.06 | 0.93 | 1.22 | 1.05 (0.87. 1.26) | > 85 vs. ≤ 85dBA | Adjusted for age and region after recalculation by the authors upon request |
| Suadicani (2012) | Composite HR | 1.03 | 0.76 | 1.39 | 1.04 (0.92. 1.19) | ≥85dBA (vocal effort) for > 0 years vs. > 85dBA (vocal effort) for 0 years | Age + lifestyle adjusted model seems the best compromise between parsimony and controlling for confounders |

Abbreviations: RR –relative risk, HR – hazard ratio. LCI/UCI – lower/upper 95% confidence interval limit.

*Table A3.4 Input data for main meta-analysis of prioritized evidence (cohort studies), Outcome: Acquired stroke (stroke incidence)*

| **Study ID** | **Risk estimate** | **Effect size** | **LCI** | **UCI** | **Pooled effect size if study excluded** | **Comparison reported in study** | **Notes** |
| --- | --- | --- | --- | --- | --- | --- | --- |
| Eriksson (2018) | HR | 1.16 | 0.82 | 1.65 | 1.07 (0.8. 1.44) | ≥ 85 vs. < 75 dBA | Age-adjusted |
| Stokholm (2013) | HR | 1.07 | 0.8 | 1.44 | 1.16 (0.82. 1.65) | ≥ 85 for 3-9 years vs. < 70 dBA | This duration of exposure is the most comparable to the other studies because the follow up in Eriksson (2018) was on average 16 years and in Gopinath (2011) – 5 years |

Abbreviations: HR – hazard ratio, LCI/UCI – lower/upper 95% confidence interval limit.

*Table A3.5 Input data for main meta-analysis of prioritized evidence (cohort studies), Outcome: Died from stroke (stroke mortality)*

| **Study ID** | **Risk estimate** | **Effect size** | **LCI** | **UCI** | **Pooled effect size if study excluded** | **Comparison reported in study** | **Notes** |
| --- | --- | --- | --- | --- | --- | --- | --- |
| Davies (2002) | Composite RR | 1.006 | 0.880 | 1.149 | 1.03 (0.90. 1.18) | ≥85dBA for > 3 years vs. > 85dBA for < 3 years |  |
| Gopinath (2011) | HR | 1.01 | 0.62 | 1.66 | 1.02 (0.92. 1.12) | Unable to hear speech vs. None; > 5 years exposure |  |
| Pettersson (2020) | RR | 1.03 | 0.9 | 1.19 | 1.01 (0.89. 1.14) | > 85 vs. ≤ 85dBA | Adjusted for age and region after recalculation by the authors upon request |

Abbreviations: RR –relative risk, HR – hazard ratio, LCI/UCI – lower/upper 95% confidence interval limit.

*Table A3.6 Input data for main meta-analysis of prioritized evidence (cohort studies), Outcome: Acquired hypertension (hypertension incidence)*

| **Study ID** | **Risk estimate** | **Effect size** | **LCI** | **UCI** | **Pooled effect size if study excluded** | **Comparison reported in study** | **Notes** |
| --- | --- | --- | --- | --- | --- | --- | --- |
| Chang (2013) | HR | 1.93 | 1.15 | 3.22 | 1.07 (0.94. 1.22) | ≥ 85 vs. < 80 dBA | Participants in the 80-85dBA category are excluded |
| Stokholm (2013) – Men | RR | 0.99 | 0.89 | 1.11 | 1.19 (0.98. 1.45) | > 85dBA for 3-9 years vs. < 70 dBA | 3-9 years of exposure was comparable to the duration of exposure in Chang (2013) (10 years), Tessier Sherman (2017) (5.9-6.6 years) and Huo Yung Kai (2018) (5 years). Group sample size was not reported (only the number of cases) and it was not possible to calculate composite effect size across all exposed groups defined by exposure duration |
| Stokholm (2013) –Women | RR | 1.05 | 0.82 | 1.34 | 1.16 (0.94. 1.42) | > 85dBA for 3-9 years vs. < 70 dBA |  |
| Tessier Sherman (2017) | Composite RR | 1.001 | 0.761 | 1.315 | 1.16 (0.95. 1.42) | ≥ 82 vs < 82 dBA | Adjusted for the potential mediator BMI. but that did not change substantially the effect compared with the unadjusted model |

Abbreviations: RR –relative risk, LCI/UCI – lower/upper 95% confidence interval limit.

***Appendix 4 Selected excluded studies and reason for their exclusion***

| **Study (Study ID)** | **Reason for exclusion** |
| --- | --- |
| Abbate 2002 | Ineligible outcome studied |
| Alfredsson 1993 | Ineligible comparator |
| Andersson 2007 | Ineligible exposure studied |
| Andren 1980 | Ineligible study type |
| Andren 1979 | Ineligible study type |
| Aro 1984 | Ineligible outcome studied |
| Avilán Rovira 2011 | Ineligible study type |
| Babisch 1990 | Ineligible outcome studied |
| Balaji 2016 | Ineligible outcome studied |
| Baranova 1989 | Ineligible comparator |
| Barbini 2017 | Ineligible comparator |
| Belli 1984 | Ineligible study type |
| Beltrame 2007 | Ineligible comparator |
| Boff 2002 | Ineligible exposure studied |
| Brahem 2019 | Ineligible study type |
| Brown 1975 | Ineligible comparator |
| Bruno 2009 | Ineligible comparator |
| Burgess 2004 | Ineligible outcome studied |
| Cavagioni 2010 | Ineligible exposure studied |
| Cayir 2018 | Ineligible study type |
| Chang 2003 | Ineligible outcome studied |
| Chang 2012 | Ineligible exposure studied |
| Chang 2011 | Ineligible study type |
| Chang 2007 | Ineligible outcome studied |
| Conceição 2006 | Ineligible study type |
| Cordeiro 1993 | Ineligible study type |
| Davies 2002 | Ineligible outcome studied |
| Deyanov 1995 | Ineligible comparator |
| Doronin 2017 | Ineligible comparator |
| Doyon 1978 | Ineligible outcome studied |

Note. This excerpt shows the first 30 studies from the list of excluded studies.

***Appendix 5 Justifications for risk of bias ratings***

*Table A5.1 Risk of bias, [Eriksson 2018b]*

| **Domain** | **Rating** | **Justification for rating** |
| --- | --- | --- |
| Bias in selection of participants into the study | Low | The study was based on a random general population sample, including a third of all men living in Gothenburg. Attrition rate during follow-up was deemed acceptable (29%). |
| Bias due to a lack of blinding of study personnel | Low | Blinding could not influence the outcome and exposure measures because this was a record-linkage study with no direct access to the study participants. |
| Bias due to exposure misclassification | Probably low | Job-exposure matrix for noise based on measurements was used, which is a standard exposure assessment approach in large-scale epidemiological studies. However, it is an indirect measure of exposure with limited accuracy on the individual-level. |
| Bias in the outcome misclassification | Low | Outcome assessment was based on a hospital discharge national register. Reference to appropriate ICD classification was made. |
| Confounding | Probably low | The study accounted for two out of three important confounders (Tier I) by statistically adjusting for age and including only male participants. It did not adjust for socioeconomic position because the exposure assessment was based on occupations, which usually comprise the socioeconomic position. |
| Bias due to incomplete outcome data | Low | No incomplete outcome data suspected as the data source was a detailed hospital discharge national register. |
| Bias due to selective reporting of outcomes | Low | All outcomes reported are consistent with the design of the study and have been reported as pre-specified in the methods section. |
| Bias due to conflict of interest | Low | We identified no conflict of interest or funding sources that could have influenced the conduct or reporting of this study. It was supported by a governmental source (Swedish Research Council) and a charitable fundraising organisation (Swedish Heart and Lung Foundation). |
| Other bias | Low | No other apparent sources of bias. |

*Table A5.2 Risk of bias, [Ising 1997]*

| **Domain** | **Rating** | **Justification for rating** |
| --- | --- | --- |
| Bias in selection of participants into the study | Low | Cases were people treated for myocardial infarction in Berlin hospitals; controls were a random sample of the male Germans with similar age drawn from the local registration office; only subjects currently working were included. Participation rate was high (91% among cases and 64% among controls), amounting to 80-85% of the source population. Obvious hints at the aim of the study were avoided, both in the questionnaires and in interviews with the patients. Differences in social class distribution between the source population and controls were taken into consideration. |
| Bias due to a lack of blinding of study personnel | Probably low | Exposure of cases was measured through a questionnaire administered by a physician who was aware of participant’s outcome status. Control subjects filled in the questionnaire themselves. We do not suspect bias because the exposure was also verified in a subsample by comparing the subjective rating to measured noise levels at the workplace. |
| Bias due to exposure misclassification | Probably high | A subjective common noise source exposure scale was used and the correlation between subjective ratings and objective noise level was studied in a small test sample of 80 subjects. However, we consider that the retrospective assessment of the level of exposure may have influenced by the experience of myocardial infarction, leading to a systematic over-estimation of noise by the MI patients. |
| Bias in the outcome misclassification | Low | Cases were people treated for myocardial infarction in Berlin hospitals, which was confirmed by the examining physician. |
| Confounding | Low | All Important confounders (Tier I) were considered in the analysis model. |
| Bias due to incomplete outcome data | Probably low | Cases were drawn from the major Berlin hospitals (80-85% of the source population) but some smaller hospitals were not included in the sampling. |
| Bias due to selective reporting of outcomes | Low | This was a case-control study with a predefined outcome; therefore, this bias cannot be at play here. |
| Bias due to conflict of interest | Low | Authors have not provided a statement on conflict of interest. However, we identified no conflict of interest or funding sources that could have influenced the conduct or reporting of this study. All study authors are affiliated with a government agency. |
| Other bias | Probably high | Potential mediators were adjusted for in the model, which could have attenuated the observed effect sizes. |

*Table A5.3 Risk of bias, [Virkkunen 2005]*

| **Domain** | **Rating** | **Justification for rating** |
| --- | --- | --- |
| Bias in selection of participants into the study | Probably low | The participants in this study were only a fraction of the original cohort sample size (Helsinki Heart Study). Only industrially employed participants were included. However, we did not suspect that inclusion/exclusion criteria, recruitment, and participation and follow-up rates different systematically between cases and controls. |
| Bias due to a lack of blinding of study personnel | Low | Participants were drawn from an originally double-blinded study. The assignment of noise exposure in the present study was not explicitly stated as being blind. However, we judge that the outcome measures as well as the exposure measures are not likely to be influenced by lack of blinding because the assessment methods were consistent across groups. |
| Bias due to exposure misclassification | Probably low | Job-exposure matrix for noise based on expert judgements and measurements was used, which is a standard exposure assessment approach in large-scale epidemiological studies. However, it is an indirect measure of exposure with limited accuracy on the individual-level. |
| Bias in the outcome misclassification | Low | Outcome assessment was based on a hospital discharge register and death register. Reference to appropriate ICD classification was made. |
| Confounding | Probably low | The study accounted for two out of three important confounders (Tier I) by statistically adjusting for age and including only male participants. It did not adjust for socioeconomic position. |
| Bias due to incomplete outcome data | Low | No incomplete outcome data suspected as the data source was a detailed hospital discharge register. The authors referenced evidence in support of those registers being accurate sources of data for epidemiological studies. |
| Bias due to selective reporting of outcomes | Low | The outcome reported is consistent with the design of the study and has been reported as pre-specified in the methods section. |
| Bias due to conflict of interest | Low | We identified no conflict of interest or funding sources that could have influenced the conduct or reporting of this study. It was supported by the Kalle Kaihari Heart Research Fund and the Scientific Foundation of the City of Tampere. |
| Other bias | Probably low | The authors adjusted the model of interest for a potential mediator (systolic blood pressure), but that did not seem to substantively reduce the effect size. |

*Table A5.4 Risk of bias, [Song 2013]*

| **Domain** | **Rating** | **Justification for rating** |
| --- | --- | --- |
| Bias in selection of participants into the study | Probably low | There was progressive reduction in the response rate across the survey cycles, but indirect evidence suggests that many actions to minimize the effect were put in place, and the potential selection effects were not differential across exposure and outcome groups. |
| Bias due to a lack of blinding of study personnel | Low | No blinding but the outcome was not likely to be influenced by lack of blinding. We judge that the self-reported outcome measures as well as the exposure status assigned on job title are not likely to be influenced by lack of blinding. |
| Bias due to exposure misclassification | Probably low | The method used for exposure assessment is similar to a job-exposure matrix system, but the process is very well described and accounts for occupational characteristics and duration of exposure. However, it is an indirect measure of exposure with limited accuracy on the individual-level. |
| Bias in the outcome misclassification | Probably high | There is indirect evidence that the outcome assessment (self-reported heart disease) method may have limited sensitivity/specificity. |
| Confounding | Low | The study accounted for all three important confounders (Tier I). Cases and controls were matched on age and sex, and the model was statistically adjusted for income. |
| Bias due to incomplete outcome data | High | Participants with missing outcome data were dropped. The number of those with unknown cardiovascular disease status(n = 2303) in the original sample was greater than the total analysis sample in the current study (n = 1326), which could have induced biologically relevant bias in effect estimate. |
| Bias due to selective reporting of outcomes | Low | This was a case-control study with predefined outcomes; therefore, this bias cannot be at play here. |
| Bias due to conflict of interest | Probably low | There is no conflict of interest statement or a disclosure of competing interests, but the study is reported in a Master of Science thesis and it is unlikely that conflict of interest exists. There is indirect evidence suggesting that the study was free of support from a company or other entity having a financial interest in the outcome of the study. |
| Other bias | Probably high | The model of interest was overadjusted for multiple potential mediators. |

*Table A5.5 Risk of bias, [Davies 2002]*

| **Domain** | **Rating** | **Justification for rating** |
| --- | --- | --- |
| Bias in selection of participants into the study | Probably low | The sample was built on employment records of softwood lumber mills to identify workers with at least 1 year of work between 1950 and 1995. Lost-to-follow (16%) up appears to have been properly managed. There is a somewhat succinct description of the sampling and recruitment process. |
| Bias due to a lack of blinding of study personnel | Low | Blinding could not influence the outcome and exposure measures because this was a record-linkage study with no direct access to the study participants. |
| Bias due to exposure misclassification | Probably low | They used a combination of measurements, interviews, hygienists' assessment and modelling. Still, non-differential exposure misclassification could be at play. |
| Bias in the outcome misclassification | Low | Outcome assessment was based on a national mortality database. Reference to appropriate ICD classification was made. |
| Confounding | Probably low | The study accounted for two out of three important confounders (Tier I) by statistically adjusting for age and including only male participants. It did not adjust for socioeconomic position. |
| Bias due to incomplete outcome data | Low | Appropriate and validated methods were applied for missing data (lost to follow-up). |
| Bias due to selective reporting of outcomes | Low | All outcomes reported are consistent with the design of the study and have been reported as pre-specified in the methods section. |
| Bias due to conflict of interest | Low | The study was supported by the Canadian Institutes for Health Research and the British Columbia Medical Services Foundation. No conflict of interest is foreseeable. |
| Other bias | Low | No other apparent sources of bias. |

*Table A5.6 Risk of bias, [Pettersson 2020]*

| **Domain** | **Rating** | **Justification for rating** |
| --- | --- | --- |
| Bias in selection of participants into the study | Probably low | This was a large-scale nationwide study among all construction workers (80% participation rate). However, about half of all original workers were excluded for the current study. This may have resulted in unknown bias but there is no evidence to suggest that inclusion/exclusion criteria, recruitment procedures, and participation and follow-up rates were inconsistent across groups. |
| Bias due to a lack of blinding of study personnel | Low | Blinding could not influence the outcome and exposure measures because this was a record-linkage study with no direct access to the study participants. |
| Bias due to exposure misclassification | Probably low | Job-exposure matrix for noise based on measurements was used, which is a standard exposure assessment approach in large-scale epidemiological studies. However, it is an indirect measure of exposure with limited accuracy on the individual-level. |
| Bias in the outcome misclassification | Low | Outcome assessment was based on death registries. Appropriate ICD coding was used to identify outcomes. |
| Confounding | Probably low | The study accounted for two out of three important confounders (Tier I) by statistically adjusting for age and including only male participants. It did not adjust for socioeconomic position. |
| Bias due to incomplete outcome data | Low | No incomplete outcome data suspected as the data source was a detailed death register. |
| Bias due to selective reporting of outcomes | Low | All outcomes reported are consistent with the design of the study and have been reported as pre-specified in the methods section. |
| Bias due to conflict of interest | Probably low | This study was funded by an insurance company (AFA Insurance), which could have interest in the outcomes of the study. Still, the authors are affiliated with a research institution and reported no conflict of interest. |
| Other bias | Low | No other apparent sources of bias. |

*Table A5.7 Risk of bias, [Gopinath 2011]*

| **Domain** | **Rating** | **Justification for rating** |
| --- | --- | --- |
| Bias in selection of participants into the study | Probably low | There was progressive reduction in the response rate across the survey cycles. However, we did not suspect that inclusion/exclusion criteria, recruitment, and participation and follow-up rates differ systematically between cases and controls. |
| Bias due to a lack of blinding of study personnel | Low | Blinding could not influence the outcome and exposure measures because this was a record-linkage study with no direct access to the study participants. |
| Bias due to exposure misclassification | High | Exposure assessment was based on a questionnaire asking about having ever been exposed to noise at the workplace and additional descriptive information. However, the self-reported measure is not completely in line with other validated questions on vocal effort. |
| Bias in the outcome misclassification | Low for IHD and stroke mortality  Probably high for stroke incidence | Mortality was confirmed by cross-matching with the Australian National Death Index. Appropriate ICD coding was used to identify outcomes.  Stroke incidence was determined through an interviewer-administered medical questionnaire (self-reported doctor diagnosis). |
| Confounding | Low | The study accounted for all three important confounders (Tier I) by statistically adjusting for age, sex and occupational prestige (proxy for socioeconomic position). |
| Bias due to incomplete outcome data | Low | No missing outcome data foreseeable. Missing subject at BMES 2 and BMES3 where anyway accounted for as non-participants. |
| Bias due to selective reporting of outcomes | Low for IHD and stroke mortality  Probably high for stroke incidence | Outcome reporting was consistent with the design of the study and as pre-specified in the methods section. Moreover, mortality status was ascertained from a national mortality register.  The authors reported the estimate for “the only significant association observed with stroke among those exposed to severe level of noise exposure for less than 1 to 5 years”. |
| Bias due to conflict of interest | Low | Study funded through government research programmes (Australian National Health and Medical Research Council and the Australian Government's Cooperative Research Centres Program). The authors declare no conflict of interest. |
| Other bias | Probably high | The model of interest was overadjusted for several potential mediators. |

*Table A5.8 Risk of bias, [McNamee 2006]*

| **Domain** | **Rating** | **Justification for rating** |
| --- | --- | --- |
| Bias in selection of participants into the study | Low | The study cohort included all industrial male workers at the two sampling sites. Controls were chosen from cohort members at the same site, matched as closely as possible on age and year of starting work, with a maximum difference of three years being allowed on these variables. We did not suspect that inclusion/exclusion criteria, recruitment and participation rates different systematically between cases and controls. |
| Bias due to a lack of blinding of study personnel | Low | In assessing noise exposure, hygienists had no access to information that could identify subjects and therefore noise assessment was blind to case/control status. |
| Bias due to exposure misclassification | Probably low | Expert assessment of working conditions were used to estimate participants’ exposure. The procedure appeared to be valid and quality control was performed. However, the estimates may have limited accuracy on the individual-level. |
| Bias in the outcome misclassification | Low | Outcome assessment was based on death registries. Appropriate ICD coding was used to identify outcomes. |
| Confounding | Probably low | The study accounted for two out of three important confounders (Tier I) by statistically adjusting for age and including only male participants. It did not adjust for socioeconomic position. |
| Bias due to incomplete outcome data | Low | No incomplete outcome data suspected as the data source was a detailed death register. All deaths among study population members were registered. |
| Bias due to selective reporting of outcomes | Low | This was a case-control study with a predefined outcome; therefore, this bias cannot be at play here. |
| Bias due to conflict of interest | Low | We identified no conflict of interest or funding sources that could have influenced the conduct or reporting of this study. It was supported by a legitimate governmental source (UK Department of Health.). The authors are affiliated with a research institution. |
| Other bias | Low | No other apparent sources of bias. |

*Table A5.9 Risk of bias, [Suadicani 2012]*

| **Domain** | **Rating** | **Justification for rating** |
| --- | --- | --- |
| Bias in selection of participants into the study | Low | We did not suspect that inclusion/exclusion criteria, recruitment and participation rates different systematically between cases and controls. Original response rate was 87% and 75% for the present study, and only 33 invalid questionnaires were identified. |
| Bias due to a lack of blinding of study personnel | Low | Blinding could not influence the outcome and exposure measures because this was a record-linkage study with no direct access to the study participants. |
| Bias due to exposure misclassification | Probably high | The exposure was measured with a question on individual perception of noise level at the work place, which may introduce differential exposure misclassification. There is anyway indirect evidence from other studies that the specific question used was validated as a reliable measure of high noise exposure. |
| Bias in the outcome misclassification | Low | Outcome assessment was based on diagnoses from official national registers. Appropriate ICD coding was used to identify outcomes. |
| Confounding | Low | The study accounted for all three important confounders (Tier I) by statistically adjusting for age and low social class and including only male participants. |
| Bias due to incomplete outcome data | Low | Invalid questionnaires were excluded. All men who had taken part in the examination were traced from registers. No incomplete outcome data suspected as the data source was a detailed death register. |
| Bias due to selective reporting of outcomes | Low | The outcome reported is consistent with the design of the study and has been reported as pre-specified in the methods section. |
| Bias due to conflict of interest | Probably low | The study was funded by a number of Foundations (King Christian X Foundation, the Danish Medical Research Council, the Danish Heart Foundation, and Else & Mogens Wedell-Wedellsborg Foundation). However, even though a statement of no conflict of interest was provided, the authors were affiliated with public research institutions and health universities, which makes competing interests unlikely |
| Other bias | Probably high | The model of interest was overadjusted for several potential mediators. |

*Table A5.10 Risk of bias, [Girard 2015]*

| **Domain** | **Rating** | **Justification for rating** |
| --- | --- | --- |
| Bias in selection of participants into the study | High | The description of the source population and the sampling process were well described and detailed, but there was a drastic reduction in sample size from 8910 in the source population to 644 in the current study. This may have introduced an unknown degree of bias related to the exposure, as many workers were excluded based on audiometric results and hearing loss status. |
| Bias due to a lack of blinding of study personnel | Low | Lack of blinding was unlikely to have introduced bias because the original sampling and audiometric testing were carried out to study the effect of noise on hearing loss not CVD. |
| Bias due to exposure misclassification | Probably high | The variable was a crude self-reported surrogate for duration of noise exposure, actual measurements were conducted only once; there was no distinction between work and leisure time exposure. The authors acknowledged that the single workplace measurement was not representative of the worker’s long-term exposure. |
| Bias in the outcome misclassification | Low | Outcome assessment was based on death certificates. Appropriate ICD coding was used to identify outcomes. |
| Confounding | Probably low | The study accounted for all three important confounders (Tier I) by matching on follow-up duration and industrial sector (proxies for age and socioeconomic position) and including only male participants. However, the assumption that age and socioeconomic position were accounted for this way was tentative. |
| Bias due to incomplete outcome data | Low | No incomplete outcome data suspected as the data source were death certificates. |
| Bias due to selective reporting of outcomes | Low | This was a case-control study with a predefined outcome; therefore, this bias cannot be at play here. |
| Bias due to conflict of interest | Low | The authors have not provided a specific funding statement, but we judge that conflict of interest was unlikely given that all study authors were affiliated with governmental or research institutions. Moreover, they made a claim denying conflicts of interest and obtained the data from governmental institutions (Quebec National Institute of Public Health and Quebec Institute of Statistics). |
| Other bias | Low | No other apparent sources of bias. |

*Table A5.11 Risk of bias, [Stokholm 2013b]*

| **Domain** | **Rating** | **Justification for rating** |
| --- | --- | --- |
| Bias in selection of participants into the study | Probably low | The study sample was half of the original size of the source population. However, we had no serious concerns that inclusion/exclusion criteria, recruitment and participation rates differed systematically between cases and controls. |
| Bias due to a lack of blinding of study personnel | Low | Blinding could not influence the outcome and exposure measures because this was a record-linkage study. |
| Bias due to exposure misclassification | Probably low | The exposure modelling was based on measured data and described in detail. Personal measurements were also carried out in a subsample for validation. Duration of exposure was also accounted for. |
| Bias in the outcome misclassification | Low | Outcome assessment was based on diagnoses in the Danish National Patient Register. Appropriate ICD coding was used to identify outcomes. |
| Confounding | Low | The study accounted for all three important confounders (Tier I). |
| Bias due to incomplete outcome data | Low | No incomplete outcome data suspected as the data source was the Danish National Patient Register. |
| Bias due to selective reporting of outcomes | Low | The outcomes reported is consistent with the design of the study and has been reported as pre-specified in the methods section. |
| Bias due to conflict of interest | Low | We identified no conflict of interest or funding sources that could have influenced the conduct or reporting of this study. It was supported by governmental sources (Danish Working Environment Research Fund and the Danish Working Environment Authority). All study authors were affiliated with research institutions. |
| Other bias | Low | No other apparent sources of bias. |

*Table A5.12 Risk of bias, [Chang 2013]*

| **Domain** | **Rating** | **Justification for rating** |
| --- | --- | --- |
| Bias in selection of participants into the study | Low | We did not suspect that inclusion/exclusion criteria, recruitment and participation rates different systematically between cases and controls. While about a quarter of the original subjects were excluded from the current study, the authors compared participants and non-participants and found no major differences in terms of cardiovascular risk factors. |
| Bias due to a lack of blinding of study personnel | Low | No blinding but the outcome based on both self-reports and measured blood pressure was not likely to be influenced by lack of blinding. Measurements of noise exposure were conducted at baseline and at follow-up in a way that would seem independent of outcome assessment. Outcome status at follow-up would be unknown at the time of baseline noise measurements. |
| Bias due to exposure misclassification | Low | Clearly detailed 8-hour time-weighted average exposure measurement method and procedure by using a technical device and adjusting for use of ear protective devices. The tasks performed by the worker were accounted for. |
| Bias in the outcome misclassification | Low | Outcome assessment was based on self-reported doctor diagnosis and standard blood pressure measurement protocol with the same criteria at baseline and follow. Definition of hypertension was consistent with WHO standard definition |
| Confounding | Low | The study accounted for all three important confounders (Tier I) by statistically adjusting for age and educational level (proxy for socioeconomic position) and including only male participants. |
| Bias due to incomplete outcome data | Low | Some subjects were excluded because of lack of results at follow-up, but the authors compared participants and non-participants and found no major differences in terms of cardiovascular risk factors, which gives us confidence that missing outcome data was not problematic. |
| Bias due to selective reporting of outcomes | Low | The outcome reported is consistent with the design of the study and has been reported as pre-specified in the methods section. |
| Bias due to conflict of interest | Low | We identified no conflict of interest or funding sources that could have influenced the conduct or reporting of this study. It was supported by governmental sources (National Science Council of Taiwan). All study authors were affiliated with research institutions and disclosed no conflict of interest. |
| Other bias | Probably high | The model was adjusted for potential mediators, which unexpectedly inflated the effect estimate compared with the unadjusted model. |

*Table A5.13 Risk of bias, [Huo Yung Kai 2018]*

| **Domain** | **Rating** | **Justification for rating** |
| --- | --- | --- |
| Bias in selection of participants into the study | Probably high | Attrition rate was 45% and comparisons of included and dropout participants revealed systematic differences in age, education and history of metabolic disorders. This could have affected patterns in the data. |
| Bias due to a lack of blinding of study personnel | Low | No blinding but the outcome based on measured blood pressure and the exposure based on a questionnaire were not likely to be influenced by lack of blinding, as the assessors were different. |
| Bias due to exposure misclassification | Probably high | The exposure was measured with a question on individual perception of noise level at the work place, which may introduce differential exposure misclassification. There is anyway indirect evidence from other studies that the specific question used was validated as a reliable measure of high noise exposure. |
| Bias in the outcome misclassification | Low | Outcome assessment was based on standard blood pressure measurement protocol. Definition of hypertension was consistent with WHO standard definition |
| Confounding | Low | The study accounted for all three important confounders (Tier I). |
| Bias due to incomplete outcome data | Low | There were only 8% of participants with missing outcome data at follow-up, which we do not see as influential. |
| Bias due to selective reporting of outcomes | Low | All outcomes reported are consistent with the design of the study and have been reported as pre-specified in the methods section. |
| Bias due to conflict of interest | Low | We identified no conflict of interest or funding sources that could have influenced the conduct or reporting of this study. It was supported by governmental sources (French National Agency for Research). All study authors were affiliated with research institutions and disclosed no conflict of interest. |
| Other bias | Probably high | The model was adjusted for potential mediators, which unexpectedly inflated the effect estimate compared with the unadjusted model. Suspected attenuation introduced by the adjustment for many mediators. |

*Table A5.14 Risk of bias, [Stokholm 2013a]*

| **Domain** | **Rating** | **Justification for rating** |
| --- | --- | --- |
| Bias in selection of participants into the study | Probably low | The study sample was half of the original size of the source population. However, we had no serious concerns that inclusion/exclusion criteria, recruitment and participation rates differed systematically between cases and controls. |
| Bias due to a lack of blinding of study personnel | Low | Blinding could not influence the outcome and exposure measures because this was a record-linkage study. |
| Bias due to exposure misclassification | Probably low | The exposure modelling was based on measured data and described in detail. Personal measurements were also carried out in a subsample for validation. Duration of exposure was also accounted for. |
| Bias in the outcome misclassification | Probably low | Outcome assessment was based on diagnoses in the Danish National Patient Register. Appropriate ICD coding was used to identify outcomes. However, unlike IHD and stroke, hypertension may go undetected, so the real prevalence may be underestimated. |
| Confounding | Low | The study accounted for all three important confounders (Tier I). |
| Bias due to incomplete outcome data | Low | No incomplete outcome data suspected as the data source was the Danish National Patient Register. |
| Bias due to selective reporting of outcomes | Low | The outcomes reported is consistent with the design of the study and has been reported as pre-specified in the methods section. |
| Bias due to conflict of interest | Low | We identified no conflict of interest or funding sources that could have influenced the conduct or reporting of this study. It was supported by governmental sources (Danish Working Environment Research Fund and the Danish Working Environment Authority). All study authors were affiliated with research institutions. |
| Other bias | Low | No other apparent sources of bias. |

*Table A5.15 Risk of bias, [Tessier-Sherman 2017]*

| **Domain** | **Rating** | **Justification for rating** |
| --- | --- | --- |
| Bias in selection of participants into the study | Probably high | The study cohort included all industrial male workers sampling sites meeting inclusion criteria. Still, only those in a job that required both noise exposure monitoring and audiometric testing were included in the current study and it is unknown whether they differed systematically from all employees in the company. The sample may be skewed towards highly exposed individuals. |
| Bias due to a lack of blinding of study personnel | Low | Blinding could not influence the outcome and exposure measures because this was a record-linkage study. |
| Bias due to exposure misclassification | Probably low | The exposure assessment method was objective and described in detail. Personal measurements were collected for each job title to construct a database. Measurements followed Occupational Safety and Health Administration (OSHA) protocol. Still, accuracy at the individual-level may be limited. |
| Bias in the outcome misclassification | Probably low | Outcome assessment was based on medical insurance claims. Appropriate ICD coding was used to identify outcomes. However, unlike IHD and stroke, hypertension may go undetected, so the real prevalence may be underestimated. |
| Confounding | Low | The study accounted for all three important confounders (Tier I) by statistically adjusting for age and socioeconomic position and including only male participants. |
| Bias due to incomplete outcome data | Low | No incomplete outcome data suspected as the data source was an official medical registry. |
| Bias due to selective reporting of outcomes | Low | The outcomes reported is consistent with the design of the study and has been reported as pre-specified in the methods section. |
| Bias due to conflict of interest | High | Study funded by grants from institutions employing some of the authors and from the company where the study was conducted, which partly covered the compensation of some of the authors through a contractual agreement. |
| Other bias | Probably low | The effect estimate was adjusted for potential mediators (body mass index and hearing); however, comparison of bivariate and adjusted models did not indicate major impact of those mediators. |

*Table A5.16 Risk of bias, [Tong 2017]*

| **Domain** | **Rating** | **Justification for rating** |
| --- | --- | --- |
| Bias in selection of participants into the study | Probably high | Inclusion criteria were strict and may have undermined the representativeness of the analysis sample compared with all workers in the factory. There was no comparison between included and excluded workers. |
| Bias due to a lack of blinding of study personnel | Low | No blinding but the cases and controls had the same inclusion and exclusion criteria. The outcome was assessed during an annual medical examination and the exposure assessment did not appear influenced by outcome status. |
| Bias due to exposure misclassification | Low | The exposure assessment method was objective taking into account the average levels of noise and the time spent in each area. 40 hours of time-weighted average levels were detected according the type of work, detention time, and work shift situation. |
| Bias in the outcome misclassification | Low | Outcome assessment was based on diagnostic criteria outlined in the US Preventive seventh report of the Joint National Committee on Detection, Evaluation and Treatment of Hypertension (JNC 7) and medical history of workers. |
| Confounding | Probably low | The study accounted for two out of three important confounders (Tier I) by supporting same mean age across cases and controls and including only male participants. It did not adjust for socioeconomic position and education years differed between those groups. |
| Bias due to incomplete outcome data | Probably low | No information on missing data and the number of workers who did not participate in the occupational physical examination. Still, we judge that this percentage was not likely to be high as the company carried out this official medical screening. |
| Bias due to selective reporting of outcomes | Low | This was a case-control study with a predefined outcome; therefore, this bias cannot be at play here. |
| Bias due to conflict of interest | Probably high | The study received funding from a provincial body; however, one of the authors was affiliated with the Tangshan Iron and Steel Group Co., Ltd plant. |
| Other bias | Probably high | Overadjusted for several mediators, which reduced the effect size. |

*Table A5.17 Risk of bias, [Kersten 2015]*

| **Domain** | **Rating** | **Justification for rating** |
| --- | --- | --- |
| Bias in selection of participants into the study | Probably high | The cases and controls were recruited from major hospitals in Berlin. Hospital controls are not optimal for a case-control study and their selection was not detailed. |
| Bias due to a lack of blinding of study personnel | Low | No blinding but the exposure assessment did not likely to be influenced by lack of blinding about outcome status, as it was based on job-title classification. |
| Bias due to exposure misclassification | Probably high | The noise variable was validated in a subsample where it correlated with measured noise levels; they accounted for long-term exposure and hearing protector use. Still, accuracy at the individual-level may be low. |
| Bias in the outcome misclassification | Low | Outcome assessment was based on a hospital records. Reference to appropriate ICD classification was made. |
| Confounding | Low | The study accounted for all three important confounders (Tier I). Cases and controls were matched on age and sex, and the authors used directed acyclic graphs to identify the most parsimonious adjustment set. |
| Bias due to incomplete outcome data | Probably low | Cases were drawn from major Berlin hospitals but some smaller hospitals were not included in the sampling. |
| Bias due to selective reporting of outcomes | Low | This was a case-control study with a predefined outcome; therefore, this bias cannot be at play here. |
| Bias due to conflict of interest | Low | Authors reported on conflict of interest and no financial support. All study authors are affiliated with a government agency. |
| Other bias | Low | No other apparent sources of bias. |
